# Maternal Broadly Neutralizing Antibodies Can Select for Neutralization-Resistant, Infant-Transmitted/Founder HIV Variants

**DOI:** 10.1128/mBio.00176-20

**Published:** 2020-03-10

**Authors:** David R. Martinez, Joshua J. Tu, Amit Kumar, Jesse F. Mangold, Riley J. Mangan, Ria Goswami, Elena E. Giorgi, Juilin Chen, Michael Mengual, Ayooluwa O. Douglas, Holly Heimsath, Kevin O. Saunders, Nathan I. Nicely, Joshua Eudailey, Giovanna Hernandez, Papa Kwadwo Morgan-Asiedu, Kevin Wiehe, Barton F. Haynes, M. Anthony Moody, Celia LaBranche, David C. Montefiori, Feng Gao, Sallie R. Permar

**Affiliations:** a Department of Molecular Genetics and Microbiology, Duke University Medical Center, Durham, North Carolina, USA; b Duke Human Vaccine Institute, Durham, North Carolina, USA; c Los Alamos National Laboratory, Los Alamos, New Mexico, USA; d Department of Surgery, Duke University Medical Center, Durham, North Carolina, USA; e Department of Medicine, Duke University Medical Center, Durham, North Carolina, USA; f Department of Pediatrics, Duke University Medical Center, Durham, North Carolina, USA; University of Pittsburgh School of Medicine

**Keywords:** bNAbs, infant-T/F virus, mother-to-child transmission, HIV, neutralization, MTCT, antibody, vertical transmission, virus

## Abstract

Each year, >180,000 infants become infected via mother-to-child transmission (MTCT) of HIV despite the availability of effective maternal antiretroviral treatments, underlining the need for a maternal HIV vaccine. We characterized 224 maternal HIV envelope (Env)-specific IgG monoclonal antibodies (MAbs) from seven nontransmitting and transmitting HIV-infected U.S. and Malawian mothers and examined their neutralization activities against nontransmitted autologous circulating viruses and infant-transmitted founder (infant-T/F) viruses. Only a small subset of maternal viruses, 3 of 72 (4%), were weakly neutralized by maternal linear V3 epitope-specific IgG MAbs, whereas 6 out of 6 (100%) infant-T/F viruses were neutralization resistant to these V3-specific IgG MAbs. We also show that maternal-plasma broadly neutralizing antibody (bNAb) responses targeting the V3 glycan supersite in a transmitting woman may have selected for an N332 V3 glycan neutralization-resistant infant-T/F virus. These data have important implications for bNAb-eliciting vaccines and passively administered bNAbs in the setting of MTCT.

## Abstract (precis)

Efforts to eliminate MTCT of HIV with antiretroviral therapy (ART) have met little success, with >180,000 infant infections each year worldwide. It is therefore likely that additional immunologic strategies that can synergize with ART will be required to eliminate MTCT of HIV. To this end, understanding the role of maternal HIV Env-specific IgG antibodies in the setting of MTCT is crucial. In this study, we found that maternal-plasma broadly neutralizing antibody (bNAb) responses can select for T/F viruses that initiate infection in infants. We propose that clinical trials testing the efficacy of single bNAb specificities should not include HIV-infected pregnant women, as a single bNAb might select for neutralization-resistant infant-T/F viruses.

## INTRODUCTION

In 2017, over 180,000 infants became HIV infected via mother-to-child transmission (MTCT) worldwide (1), with the majority of infections occurring in developing countries. When optimally employed, antiretroviral therapy (ART) during pregnancy can reduce rates of MTCT of HIV to below 1% (2). Yet, several challenges remain for the elimination of pediatric HIV infections, including late initiation of ART therapy and ART-associated toxicities in infants, the lack of universal HIV testing of pregnant women, HIV acquisition in pregnant and breastfeeding women, and poor maternal adherence to ART therapy throughout pregnancy and during breastfeeding (3). Additional strategies that work synergistically with maternal ART therapy to reduce the risk of MTCT, such as a maternal HIV vaccine, will be required to completely eliminate pediatric HIV infections.

In the absence of any interventions during pregnancy or near the time of delivery, less than half of infants born to HIV-infected women become infected, suggesting a possible involvement of maternal protective immune factors (4–6). Maternal immune factors that contribute to this partial protection may include maternal neutralizing antibodies (NAbs) that are placentally transferred to the infant throughout gestation. Clear evidence demonstrates that efficiently transferred maternal antibodies across the placenta are protective against infectious neonatal pathogens, such as influenza, measles, pertussis, and tetanus (7–14). However, whether maternal HIV envelope (Env)-specific IgG responses can protect the fetus and newborn against vertical transmission of HIV remains controversial. Several studies suggest that nontransmitting HIV-infected women have Env-specific IgG responses that are associated with protection compared to those of transmitting mothers (15–18). In contrast, other studies reported higher levels and neutralization breadth of NAbs in transmitting than in nontransmitting HIV-infected women (19–21). The lack of agreement among these findings may be due to differences in study design and inadequate control for factors known to impact MTCT risk, such as maternal-plasma viral load, peripheral blood CD4^+^ T cell count, and maternal ART during pregnancy, potentially confounding the role of maternal Env-specific antibody responses. A better understanding of the role of maternal HIV-neutralizing antibodies is needed to help guide immune-based interventions to further reduce MTCT.

Our group previously defined maternal humoral correlates of protection against peripartum HIV transmission in a large cohort of 248 HIV-infected, ART-naive U.S. mothers from the Women and Infant Transmission Study (WITS) (17). In the WITS cohort, maternal CD4 binding site (CD4bs)-blocking IgG responses, tier 1 virus-neutralizing antibody responses, and variable loop 3 (V3)-specific IgG binding responses were associated with reduced MTCT risk (17). Remarkably, V3-specific IgG monoclonal antibodies (MAbs) obtained from a nontransmitting mother neutralized a large proportion of nontransmitted autologous viruses in the mother’s plasma, suggesting that these commonly elicited V3-specific IgG responses may impede MTCT through autologous-virus neutralization (17). A deeper analysis of these potentially protective maternal V3-specific IgG responses mapped their specificity to the C-terminal region of the V3 loop (16). A separate study of chronically HIV-infected adults reported that linear V3-specific IgG responses in plasma strongly neutralized a large proportion of circulating autologous viruses in plasma and also selected for neutralization-resistant variants that repopulated the virus pool, suggesting that linear V3-specific IgG responses can exert strong immune pressure on autologous plasma variants (22). HIV can escape from host autologous-virus-neutralizing antibodies via several mechanisms, which include shifting Env glycans, epitope sequence alteration, and conformational masking of proximal and distal epitopes (23, 24). We recently reported that infant-transmitted founder (infant-T/F) viruses are more neutralization resistant to paired maternal-plasma viruses than to nontransmitted maternal-plasma viruses, indicating that a potential mechanism by which infant-T/F viruses are selected is via the escape from maternal-plasma autologous-virus-neutralizing antibodies (25). A better understanding of the evolution dynamics of maternal neutralizing antibodies and maternal autologous viruses, which is the precursor pool of the infant-T/F viruses, will be important to design immunologic strategies to block the vertical transmission of HIV.

Up to 20% of HIV-infected adults develop broadly neutralizing antibodies (bNAbs) during the course of infection (26). bNAbs target vulnerable regions of the HIV Env protein, including V1V2 glycans, V3 glycans, the membrane-proximal external region (MPER), and the CD4 binding site (27, 28). bNAbs can exert strong immune pressure on viruses and select escape mutants (29–31). In HIV-infected individuals, the coevolution of HIV Env and V3 glycan-targeting bNAbs can result in escape mutations in the N332 glycan supersite (32). In the setting of MTCT of HIV, a recent study found that transmitting HIV-infected women had higher plasma bNAb activity than nontransmitting women (19). However, this study did not define whether maternal-plasma broad neutralization activity selected for bNAb-resistant T/F viruses in infants. Thus, it remains unclear whether maternal-plasma bNAbs can protect against MTCT or whether they select for bNAb-resistant infant-T/F viruses. As ongoing clinical trials explore the use of HIV-specific bNAbs as passively administered therapeutics in combination with ART treatments in HIV-infected individuals, it is critical to define mechanisms of HIV escape from bNAbs (33–36). It will also be crucial to assess whether passively administered bNAbs may be used in combination with ART therapy in HIV-infected pregnant women to further reduce MTCT or whether this strategy puts the infant at higher risk of acquiring a bNAb-resistant variant, leading to the contraindication of the use of passively administered bNAbs during pregnancy. Moreover, as a major focus of the HIV vaccine field is to design immunogens that can elicit bNAbs (30, 37–41), it will also be important to define the impact of these vaccine strategies on vertical virus transmission.

In this study, we characterized the HIV Env-specific B cell repertoire from three nontransmitting and four transmitting mothers and defined the neutralizing activities of their Env-specific MAbs against circulating autologous viruses. In addition, we defined the infant-T/F viruses and characterized their neutralization sensitivity to paired maternal plasma samples and monoclonal antibodies. Finally, we also examined whether maternal-plasma bNAb responses in transmitting HIV-infected women select for neutralization-resistant infant-T/F viruses. Our findings reveal novel insights into the role of maternal HIV Env-specific neutralizing antibodies and suggest that passively administered bNAbs in the setting of pregnancy may select for escape variants that initiate infant HIV infection.

## RESULTS

### Clinical characteristics of nontransmitting and transmitting HIV-infected U.S. and Malawian women.

Two HIV-infected U.S. women in this study were enrolled in the WITS cohort between 1992 and 1993, prior to the use of ART prophylaxis in pregnancy as the standard of clinical care. Five pregnant HIV-infected Malawian women in this cohort, enrolled in 2007 to 2008, received a single dose of nevirapine at delivery. Of these HIV-infected women, three were nontransmitting women (one clade B HIV-infected U.S. woman and two clade C HIV-infected Malawian women). The remaining four were transmitting women, including one clade B HIV-infected U.S. woman and three clade C HIV-infected Malawian women. Nontransmitting U.S. and Malawian women had a median CD4^+^ T cell count of 406 cells/mm^3^ and a median viral load of 58,915 copies/ml, and transmitting women had a median CD4^+^ T cell count of 281 cells/mm^3^ and a median viral load of 78,730 copies/ml (see Table S1 in the supplemental material). We selected three nontransmitting HIV-infected and four transmitting HIV-infected women based on adequate plasma and viable peripheral blood mononuclear cell (PBMC) samples available from delivery (see Materials and Methods for detailed selection criteria). Of the four HIV-infected infants, two (infants 155.1 and 0616) became infected perinatally, and the other two (infants 3915 and 9112) were infected *in utero* (Table S1). All infants were born by vaginal delivery except for one HIV-infected infant (155.1), who was born by Caesarean section, and one HIV-exposed uninfected infant (193.1), for whom the delivery mode is unknown.

10.1128/mBio.00176-20.9 TABLE S1 Maternal clinical characteristics, numbers of amplified infant and maternal Env sequences, and time points of collection of maternal and infant plasma and PBMC samples of nontransmitting and transmitting HIV-infected U.S. and Malawian women. Download Table S1, PDF file, 0.02 MB. Copyright © 2020 Martinez et al. 2020 Martinez et al. This content is distributed under the terms of the Creative Commons Attribution 4.0 International license.

### Phylogenetic relationships of maternal and infant *env* gene sequences.

Infant plasma samples were used to amplify a total of 121 full-length envelope (*env*) gene single-genome amplicon (SGA) sequences (Table S1). Two peripartum-infected infants (155.1 and 0616) and one *in utero*-infected infant (9112) were infected with a single transmitted founder (T/F) virus, whereas one *in utero*-infected infant, 3902, was infected with at least three genetically distinct T/F viruses (Table S1). We calculated the timing of infection in each infant by estimating the age in days of the most recent common ancestor (MRCA) using the Poisson-Fitter tool (42). Infant 0616 was estimated to have been infected before or around the time of delivery, whereas infant 155.1 was estimated to have been infected at delivery (Table S1). In contrast, the *in utero-*infected infants were estimated to have been infected in the last month of gestation based on their MRCA (Table S1).

Totals of 130 and 104 full-length *env* sequences were obtained from the plasma of four transmitting and three nontransmitting women, respectively, near the time of delivery or at delivery (Table S1). Phylogenetic tree and Highlighter plot analyses of the maternal and infant *env* sequences showed that infant viruses had low genetic diversity but that their paired maternal viruses had high genetic diversity (Fig. S1 and S2), typically with two or more plasma *env* variant lineages, as expected in chronic infections. Using an algorithm described previously (25), we selected 6 to 17 representative plasma *env* variants from each nontransmitting and transmitting woman (a total of 23 and 51 *env* variants from nontransmitting and transmitting women, respectively) and used these variants for pseudovirus production.

10.1128/mBio.00176-20.1 FIG S1 HIV *env* single-genome amplicons isolated from transmitting HIV-infected women and their paired infants. (A to D) Neighbor-joining trees and Highlighter plots of peripartum-transmitting U.S. and Malawian mother-infant pairs and of *in utero*-transmitting Malawian mother-infant pairs. One T/F virus was identified for infants born to transmitting women 155.1, 0601, and 9105, whereas three T/F viruses were identified for the infant born to transmitting woman 3902. Infant amplicons are shown as red squares, and maternal amplicons are shown as blue squares in the neighbor-joining trees. Red tick marks denote nonsynonymous amino acid mutations, and green tick marks denote synonymous amino acid mutations in the Highlighter plots. Download FIG S1, PDF file, 1.2 MB. Copyright © 2020 Martinez et al. 2020 Martinez et al. This content is distributed under the terms of the Creative Commons Attribution 4.0 International license.

10.1128/mBio.00176-20.2 FIG S2 HIV *env* single-genome amplicons isolated from nontransmitting HIV-infected women. (A to C) Neighbor-joining trees and Highlighter plots of nontransmitting U.S. and Malawian women. Maternal amplicons are shown as blue boxes in the neighbor-joining trees. Red tick marks denote nonsynonymous amino acid mutations, and green tick marks denote synonymous amino acid mutations in the Highlighter plots. Download FIG S2, PDF file, 0.5 MB. Copyright © 2020 Martinez et al. 2020 Martinez et al. This content is distributed under the terms of the Creative Commons Attribution 4.0 International license.

### Isolation of Env-specific IgG MAbs from nontransmitting and transmitting HIV-infected women.

We selected MconSgp120 as the Env “bait” to isolate antigen-specific memory B cells, as this reagent most closely aligned with HIV clade C and clade B viruses, which were the clades with which the Malawian Center for HIV/AIDS Vaccine Immunology 009 (CHAVI009) and U.S. WITS pregnant women were primarily infected. Using antigen-specific memory B cell sorting and recombinant MAb production, a total of 133 HIV Env-specific IgG MAbs were generated from the three nontransmitting HIV-infected mothers (Table S2), and 91 HIV Env-specific IgG MAbs were generated from the four transmitting HIV-infected mothers (Table S2). This Env-specific IgG B cell repertoire analysis revealed diverse antigen specificities, including variable loops 1 and 2 (V1V2), variable loop 3 (V3), and CD4 binding site (CD4bs)-specific IgG MAbs in both nontransmitting and transmitting mothers (Table S2). Mapping of the Env-specific IgG MAbs revealed 15 (11.4%) V1V2-specific, 22 (16.8%) V3-specific, and 16 (12.2%) CD4bs-specific IgG MAbs of 133 total Env-specific MAbs from three nontransmitting women. Similarly, 10 (11%) V1V2-specific, 15 (16.5%) V3-specific, and 24 (26.4%) CD4bs-specific MAbs were characterized from a total of 91 Env-specific MAbs from four transmitting women. Of the 133 Env-specific IgG MAbs from nontransmitting women, 129 (98.5%) were IgG1 and 2 (1.5%) were IgG3. Similarly, of the 91 Env-specific IgG MAbs from transmitting HIV-infected women, 87 (95.6%) were IgG1 and 4 (4.4%) were IgG3 (Table S2), consistent with the predominance of Env-specific IgG1 subclass responses in HIV-infected pregnant women (43).

10.1128/mBio.00176-20.10 TABLE S2 HIV Env binding profile of small-scale transient transfection of monoclonal IgG isolated from nontransmitting and transmitting HIV-infected U.S. and Malawian women. Download Table S2, XLSX file, 0.1 MB. Copyright © 2020 Martinez et al. 2020 Martinez et al. This content is distributed under the terms of the Creative Commons Attribution 4.0 International license.

### Affinity maturation and immunogenetic characteristics of Env-specific IgG MAbs obtained from nontransmitting and transmitting women.

**FIG 1:**
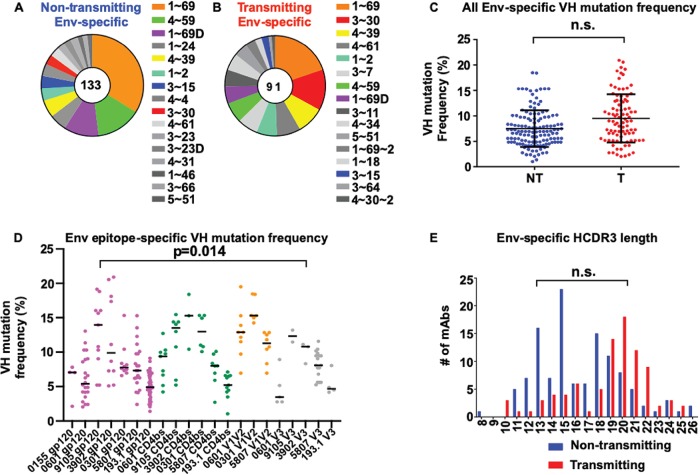
Immunogenetic characteristics of Env-specific IgG MAbs isolated from HIV-infected U.S. and Malawian women. (A and B) V_H_ gene usage of Env-specific IgG MAbs isolated from nontransmitting and transmitting women. (C) V_H_ somatic hypermutation frequency of HIV Env-specific IgG MAbs isolated from nontransmitting (blue) (NT) and transmitting (red) (T) women. n.s., differences were not statistically significant. (D) V_H_ somatic hypermutation frequency of gp120, V1V2, V3, and CD4 binding site-specific IgG MAbs. V_H_ somatic hypermutation frequencies were statistically significantly different across different epitope specificities (ANOVA *P* = 0.014). (E) HCDR3 amino acid length of Env-specific IgG MAbs. The *P* value in panel D compares the V_H_ somatic hypermutation frequency of V1V2 to those of V3, the CD4 binding site, and gp120-specific IgG MAbs in both nontransmitting and transmitting women.

We found no variable heavy (V_H_)-chain gene usage differences in Env-specific IgG MAbs obtained from either nontransmitting or transmitting women (Fig. 1A and B). V_H_1–69 was the most frequently occurring allelic variant of Env-specific IgG in both nontransmitting and transmitting mothers. To examine the V_H_ mutation differences among the Env-specific IgG MAbs obtained from nontransmitting and transmitting mothers, we utilized a previously described permutation test which accounts for multiple measurements (i.e., multiple MAbs obtained per patient) from each patient (25). We detected no significant difference in the V_H_ somatic mutation levels of Env-specific IgG MAbs obtained from nontransmitting and transmitting HIV-infected mothers (*P* = 0.22) (Fig. 1C). However, the permutation test does not account for possible differences in epitope specificity and sampling time. In order to account for these additional variables, we modeled these data using a random-effect generalized linear model (GLM) which also accounts for multiple measures within each patient (the random effect). This approach confirmed our finding from the permutation test of no statistical difference in V_H_ somatic-mutation rates between transmitting and nontransmitting mothers. However, the epitope specificity was a significant predictor of V_H_ somatic-mutation rates (*P* = 0.014, analysis of variance [ANOVA] test) (Fig. 1D). In particular, the GLM model showed that the V_H_ chain somatic hypermutation of V1V2-specific IgG MAbs was higher than those of gp120, V3, and CD4bs-specific IgG MAbs, independently of transmission status (*P* = 5 × 10^−12^, 2 × 10^−9^, and 6 × 10^−8^, respectively) (Fig. 1D). Finally, there was no difference in the heavy-chain complementarity-determining region 3 (HCDR3) lengths of the Env-specific IgG MAbs in nontransmitting HIV-infected mothers from those of transmitting mothers (Fig. 1E).

### Tier 1 virus neutralization activity of Env-specific MAbs obtained from nontransmitting and transmitting HIV-infected women.

**FIG 2:**
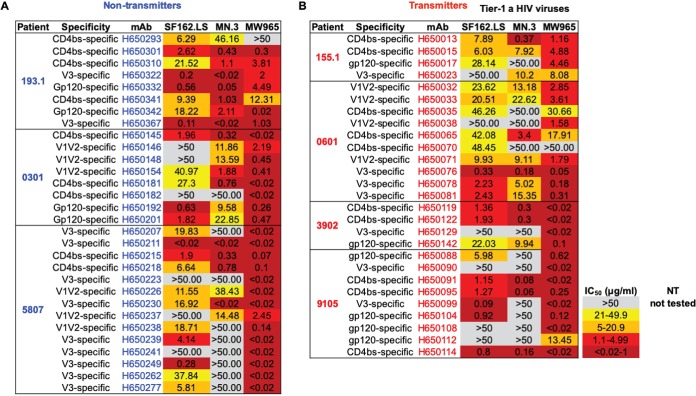
Neutralization of HIV clade B and clade C tier 1a viruses by maternal-Env-specific IgG MAbs produced by large scale. (A and B) HIV Env-specific IgG MAbs isolated from nontransmitting (blue) and transmitting (red) women against SF162.LS, MN.3, and MW965.

To capture a representative snapshot of maternal HIV Env-specific IgG antibodies, we selected the most genetically diverse MAbs as defined by the following criteria: distinct V_H_ gene usage, different somatic-mutation frequencies, different HCDR3 lengths, and distinct Env specificities (i.e., V3 versus V1V2). The results of these selection criteria yielded 30 (22%) and 27 (29%) Env-specific IgG MAbs from nontransmitting and transmitting U.S. and Malawian women, respectively, for large-scale production. We examined the neutralization activity of IgG MAbs of distinct antigen specificities against clade B and clade C tier 1A viral isolates. We observed strong neutralization activities against SF162.LS, MN.3, and MW965 among the V1V2, V3, and CD4bs-specific IgG MAbs in both nontransmitting and transmitting women (Fig. 2A and B), which was notable given that maternal linear V3 epitope-targeting MAbs with tier 1 virus-neutralizing activity have previously been associated with reduced MTCT risk (17).

### Linear V3 epitope-specific IgG MAbs obtained from nontransmitting and transmitting women mediate low levels of autologous-virus neutralization.

Overall, levels of maternal-plasma autologous-virus neutralization activity against circulating nontransmitted viruses were similar in this small cohort of nontransmitting and transmitting women. The plasma-neutralizing responses in nontransmitting women against circulating autologous viruses was overall low, with a median 50% inhibitory dilution (ID_50_) of 31 (range, <20 to 62) (Fig. S3). The neutralizing responses in the plasma of transmitting women to circulating autologous viruses were similarly low, with a median ID_50_ of 51 (range, <20 to 212) (Fig. S4). There was no difference in the neutralization sensitivity of infant T/F viruses compared to nontransmitted maternal circulating variants. However, given the limited sample size, we may be underpowered to detect differences in neutralization activity in this small group of patients.

10.1128/mBio.00176-20.3 FIG S3 Plasma and Env-specific IgG autologous-virus neutralization activity against nontransmitted maternal viruses in nontransmitting HIV-infected mothers. HIV Env-specific IgG MAbs isolated from nontransmitting mothers are shown in the blue font. Download FIG S3, PDF file, 0.02 MB. Copyright © 2020 Martinez et al. 2020 Martinez et al. This content is distributed under the terms of the Creative Commons Attribution 4.0 International license.

10.1128/mBio.00176-20.4 FIG S4 Plasma and Env-specific IgG autologous-virus neutralization activity against nontransmitted maternal viruses and their paired infant-T/F virus in transmitting HIV-infected mothers. HIV Env-specific IgG MAbs isolated from transmitting mothers are shown in the red font. The variants that were closest to the most genetically distinct maternal variants in relation to each paired infant-T/F virus are arranged from left to right, as shown by the arrow. Download FIG S4, PDF file, 0.03 MB. Copyright © 2020 Martinez et al. 2020 Martinez et al. This content is distributed under the terms of the Creative Commons Attribution 4.0 International license.

We previously reported that both maternal-plasma tier 1 virus-neutralizing responses and linear V3 epitope-specific IgG binding responses were associated with a reduced risk of MTCT (17, 43). Thus, we examined the autologous-virus-neutralizing activity of Env-specific monoclonal IgG responses in nontransmitting and transmitting women. Neither V1V2, CD4 binding site, nor gp120-specific IgG MAbs obtained from nontransmitting or transmitting women had autologous-virus-neutralizing activity (Fig. S3 and S4). In contrast, linear V3 epitope-specific IgG MAbs neutralized a very small proportion (3 of 72 [4%]) of nontransmitted maternal autologous viruses (Fig. S3 and S4). While the neutralizing activity of linear V3 epitope-specific IgG against maternal autologous viruses was overall limited, this agrees with previous studies in which maternal linear V3-specific IgG responses neutralized nontransmitted maternal autologous viruses (17, 43). In nontransmitting Malawian woman 5807, five (55%) of nine linear V3 epitope-specific IgG MAbs neutralized one (14%) of seven maternal autologous viruses (Fig. S3). Similarly, in *in utero*-transmitting women 9105 and 3902, linear V3 epitope-specific IgG MAb H650099 neutralized 1 (7%) of 13 maternal circulating nontransmitted viruses and linear V3 epitope-specific IgG MAb H650129 neutralized 1 (5%) of 17 of nontransmitted maternal autologous viruses, respectively (Fig. S4). As with the majority of the nontransmitted maternal viruses, the paired infant-T/F viruses identified in infants born to *in utero*-transmitting HIV-infected Malawian women 9105 and 3902 were neutralization resistant to the maternal linear V3 epitope-specific IgG MAbs H650099 and H650129 (Fig. S4). In contrast, we did not observe autologous-virus neutralization by linear V3 epitope-specific IgG in nontransmitting women 193.1 and 0301 or in transmitting women 155.1 and 0601 (Fig. S3 and S4). Together, these findings suggest that linear V3-specific IgG MAbs weakly and sporadically neutralize paired autologous maternal viruses in some HIV-infected women. However, an alternative explanation to this low level of autologous-virus neutralization activity in these mothers may be that these specific maternal viruses are generally more neutralization sensitive.

We previously described that potentially protective maternal V3-specific IgG responses target the C-terminal region of the V3 loop in clade B HIV-infected women (43). Thus, we examined the fine epitope specificity of maternal V3-specific IgG MAbs with autologous-virus-neutralizing activity using a library of N- and C-terminal mutant V3 peptides (Fig. S5A). Linear V3 epitope-specific IgG MAb H650367 obtained from nontransmitting woman 5807 showed strong binding activity to the wild-type V3.B peptide mutant but lower binding activity to N-terminal V3 peptide mutants V3.B K305Q I307T H308T and V3.B K305A, suggesting that amino acid residue K305 modulates its binding activity (Fig. S5B). In contrast, linear V3 epitope-specific IgG MAb H650099 obtained from transmitting woman 9105 had strong binding activity to V3.C and V3.B, and its binding activity was ablated against C-terminal V3 mutant peptides V3.B F317A A319K D322A, V3.B F317L A319T D322R, and V3.B F317A, suggesting that amino acid residue F317 was required for the binding activity (Fig. S5C). The binding activity of V3-specific IgG MAb H650129 obtained from transmitting woman 3902 was ablated by N-terminal K305Q I307T H308T and K305A I307A mutations and C-terminal F317A A319K D322A, F317L A319T D322R, F317A, and A319K mutations, suggesting that H650129 requires both N- and C-terminal amino acid residues for binding (Fig. S5D). All together, these findings suggest that both N- and C-terminal V3 loop amino acid residues mediate the binding of maternal V3-specific IgG MAbs with the ability to neutralize autologous viruses.

10.1128/mBio.00176-20.5 FIG S5 Fine-epitope specificity of maternal linear V3-specific IgG MAbs with autologous-virus-neutralizing activity. (A) Amino acid sequence alignment of V3 peptide mutant library. (B to D) Fine specificity of V3-specific IgG MAbs isolated from a nontransmitting woman and from two transmitting women. High binding strength is shown in black, medium binding strength is shown in orange, and low binding strength is shown in red. V3-specific IgG MAbs were tested at 5 μg/ml and were serially diluted threefold. V3 loop amino acid residues important for binding for each V3-specific IgG MAb are shown in blue boxes. Download FIG S5, PDF file, 0.1 MB. Copyright © 2020 Martinez et al. 2020 Martinez et al. This content is distributed under the terms of the Creative Commons Attribution 4.0 International license.

### The neutralization breadth of maternal-plasma responses in nontransmitting and transmitting HIV-infected women.

**FIG 3:**
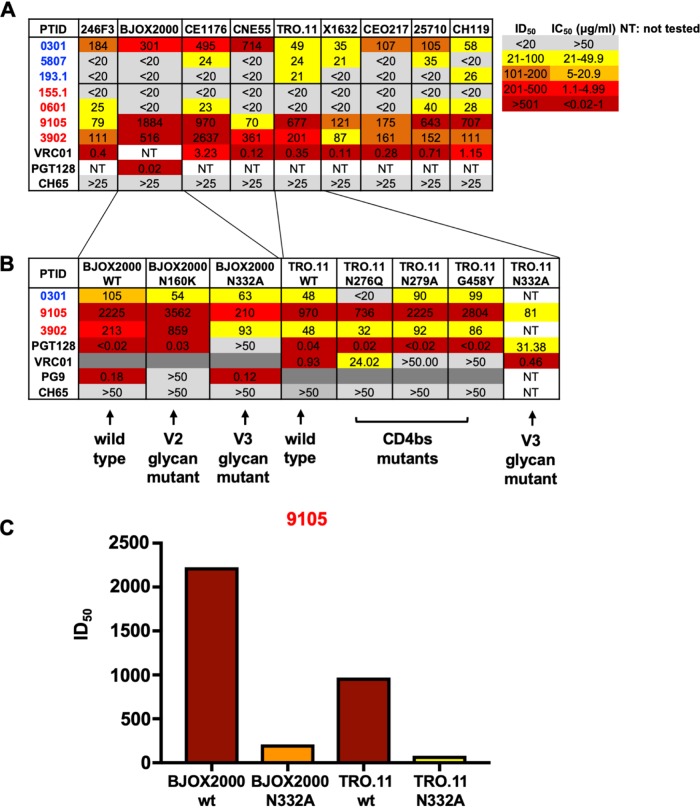
The breadth and specificity of plasma neutralizing responses in nontransmitting and transmitting HIV-infected women. (A) Plasma neutralizing antibody responses against the nine-virus global panel in nontransmitting (blue) and transmitting (red) women. (B) Mapping of plasma broadly neutralizing responses in nontransmitting and transmitting HIV-infected women with broad neutralization against V2 glycan, V3 glycan, and CD4bs mutant viruses that knocked out bNAb activity. (C) Neutralization activity of transmitting HIV-infected woman 9105 against the wild-type BJOX2000, BJOX2000 N332A mutant, wild-type TRO.11, and TRO.11 N332A mutant viruses. PTID, patient identifier; WT and wt, wild type.

A recent study that examined the neutralization breadth in clade C HIV-infected women found that transmitting mothers had significantly more broadly neutralizing plasma responses than nontransmitting mothers (19). However, other cohort studies did not replicate these findings (17, 44). Thus, it remains unclear if maternal-plasma bNAb responses are protective against vertical transmission or if they are a transmission risk factor. While we did not identify any bNAbs in the B cell repertoire analysis at the monoclonal antibody level, we characterized the plasma bNAb responses in this small cohort of nontransmitting and transmitting HIV-infected women. We assessed maternal-plasma bNAb activity by measuring neutralization breadth against a multiclade panel of HIVs that is representative of antigenic and genetic global diversity (Fig. 3A) (45). One (0301) of three nontransmitting HIV-infected women had neutralization breadth against at least seven (77%) of nine global-panel HIVs (Fig. 3A), and two (9105 and 3902) of the four transmitting HIV-infected women similarly had neutralization breadth against at least seven (77%) of nine HIVs from the global panel. To map the specificity of maternal-plasma bNAbs in nontransmitting and transmitting women with the ability to neutralize the majority of the global-panel viruses, we tested their plasma samples against a panel of mutant viruses to define the neutralization specificities. We utilized (i) the BJOX2000 N160K mutant, which is diagnostic for V2 glycan-targeting bNAbs, BJOX2000 N332A and TRO.11 N332A mutants, which are diagnostic for V3 glycan-targeting bNAbs, TRO.11 N276Q, TRO.11 N279A, and TRO.11 G458Y mutant viruses, which are diagnostic of CD4bs-targeting bNAbs, and (ii) Ce1176 N88A and Ce1176 N625A, which are diagnostic of gp120/gp41 interface glycan-targeting bNAbs. We failed to detect a reduction in the neutralization activity against BJOX2000 N160K, BJOX2000 N332A, TRO.11 N276Q, TRO.11 N279A, TRO.11 G458Y, Ce1176 N88A, and Ce1176 N625A mutants compared to the neutralization activity against wild-type BJOX2000, TRO.11, and Ce1176 viruses in nontransmitting woman 0301 or transmitting woman 3902, suggesting the absence of V2 glycan-targeting, V3 glycan-targeting, CD4 binding site-targeting, and gp120/gp41 interface glycan-targeting plasma bNAb responses (Fig. 3B; see also Fig. S6). Yet in *in utero*-transmitting woman 9105, we observed a >10-fold reduction in neutralization activity against BJOX2000 N332A compared to that against the wild-type BJOX2000 virus, as well as a 12-fold reduction in neutralization activity against TRO.11 N332A compared to that against the wild-type TRO.11 virus (Fig. 3B and C), suggesting the presence of bNAbs with V3 glycan specificity.

10.1128/mBio.00176-20.6 FIG S6 Gp120/gp41 interface glycan bNAb mapping in transmitting HIV-infected woman 3902. (A) Woman 3902 plasma was tested against the gp120-gp41 interface glycan-ablating mutant viruses Ce1176 N88A, Ce1176 N625A, CD4 binding site glycan-ablating mutant virus Ce1176 N276Q, and V3 glycan-ablating mutant virus Ce1176 N332A. Download FIG S6, PDF file, 0.01 MB. Copyright © 2020 Martinez et al. 2020 Martinez et al. This content is distributed under the terms of the Creative Commons Attribution 4.0 International license.

**FIG 4:**
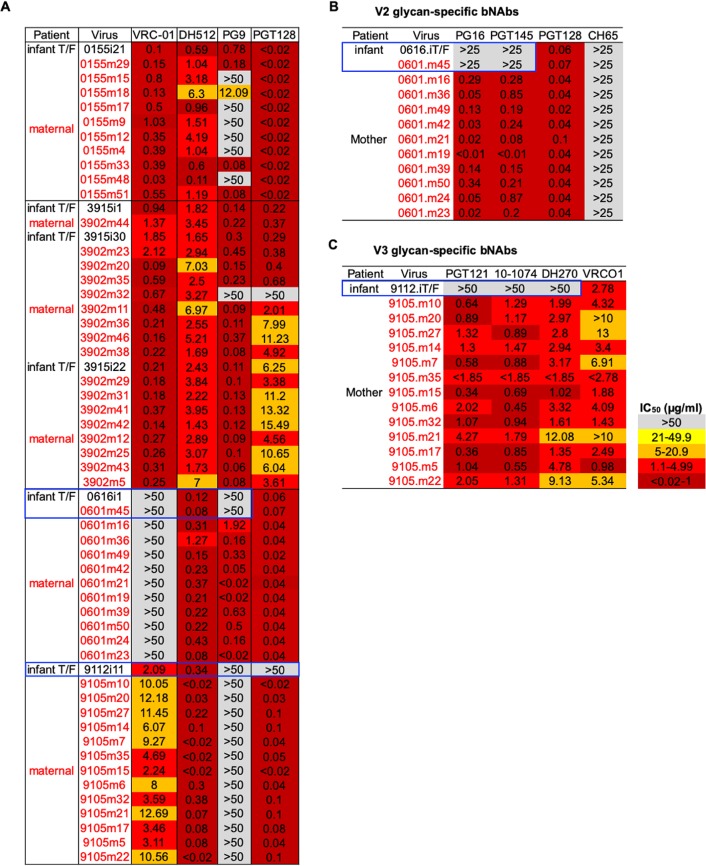
Neutralization sensitivity of circulating nontransmitted maternal viruses and infant-T/F viruses to broadly neutralizing antibodies. (A) Neutralization sensitivity to VRC01 CD4bs-specific IgG bNAb, DH512 MPER-specific IgG bNAb, PG9 V2 glycan-specific IgG bNAb, and PGT128 V3 glycan-specific IgG bNAb. (B) Differential neutralization sensitivities of infant-T/F and maternal circulating Env variants to PG16 and PGT45 V2 glycan-targeting bNAbs. (C) Differential neutralization sensitivities of infant-T/F and maternal circulating Env variants to PGT121, 10-1074, and DH270 V3 glycan-specific IgG bNAbs. Maternal Env variants from transmitting women are shown in the red font, and infant-T/F viruses are in the black font. The variants that are closest to the most genetically distinct maternal variants are arranged top to bottom with respect to their paired infant-T/F viruses.

We detected a uniform neutralization-resistant pattern in mother-infant pair 9105 in nontransmitted maternal variants and in the infant-T/F virus to V2 glycan-targeting bNAb PG9 (Fig. 4A). Interestingly, PG9 neutralization-resistant maternal variants of pair 9105 all harbored amino acid residue 732G (Fig. S8A), which was previously reported to confer neutralization resistance to PG9 (46). Similarly, transmitting HIV-infected mother 0155 had nontransmitted maternal variants that were differentially neutralization resistant to PG9 (Fig. 4A). We looked at positions in the alignment that have previously been reported in the literature to be associated with neutralization sensitivity to PG9 and observed that nontransmitted maternal variants 0155m15, 0155m18, 0155m17, 0155m9, 0155m12, 0155m4, and 0155m33 all shared 3 amino acid residues, 33K, 169V, and 633R, that were found to confer resistance to PG9 (Fig. S8B) (47). Similarly, the nontransmitted maternal 0155m48 variant also had PG9 neutralization resistance-conferring mutations 644T and 775L. However, 0155m33 and 0155m51 also shared the 644T and 775L mutations yet were sensitive to PG9, suggesting that additional amino acid residues beyond 644T and 775L may confer the PG9 neutralization-resistant phenotype observed in 0155m48 (Fig. S8B).

In order to measure whether some amino acids across all maternal Env alignments were associated with changes in neutralization profiles against VRC01 and PG9, we used the Los Alamos National Laboratory (LANL) tool GenSig (47; https://www.hiv.lanl.gov/content/sequence/GENETICSIGNATURES/gs.html), which identifies potential genetic signatures after phylogenetically correcting for common ancestry (see Materials and Methods). Given the limited sample size, we could find only marginally significant associations. However, two sites in particular were potentially interesting given that they have previously been reported to be associated with changes in neutralization sensitivity to either PG9 or VRC01. We found amino acid K166 to be associated with resistance to PG9 after phylogenetic correction (*P* = 0.042, *q* = 0.196), in agreement with similar findings from Bricault et al. (47). Similarly, we found G269 to be associated with resistance to VRC01 after phylogenetic correction (*P* = 0.029, *q* = 0.135). In the work of Bricault et al., G269 was found to be associated with increased sensitivity to other CD4bs Abs, while D269 was found to confer resistance to VRC01.

### Neutralization sensitivity of infant-T/F viruses and nontransmitted maternal viruses to heterologous broadly neutralizing antibodies.

Several studies have established that infant-T/F viruses are not uniformly neutralization resistant to bNAbs compared to nontransmitted maternal viruses (25, 48, 49); yet, the report of higher risk of transmission in the presence of broad maternal-plasma responses raises the question of whether bNAbs present in maternal plasma select for neutralization-resistant viruses, which has important implications for passive bNAb strategies in the setting of MTCT. We evaluated the neutralization sensitivity of the six infant-T/F viruses and their paired nontransmitted maternal viruses from this cohort to CD4bs-targeting antibody VRC01, membrane-proximal external region (MPER)-targeting antibody DH512, V2 glycan-targeting antibody PG9, and V3 glycan-targeting antibody PGT128. All infant-T/F viruses and their closest maternal variant were neutralization sensitive to DH512, and most variants were neutralization sensitive to VRC01 (Fig. 4A). Infant-T/F viruses and their closest nontransmitted maternal viruses for mother-infant pairs 155.1 and 3902 were similarly neutralization sensitive to PG9 (Fig. 4A). Moreover, for mother-infant pair 155.1, some of the maternal variants were neutralization sensitive to PG9, whereas other maternal variants and the infant-T/F virus were neutralization resistant (Fig. 4A). Interestingly, for mother-infant pair 0601, both the infant-T/F virus and the closest nontransmitted maternal virus were neutralization resistant to PG9, whereas the more phylogenetically distant nontransmitted maternal viruses were uniformly neutralization sensitive to PG9, suggesting the transmission of a bNAb-resistant virus (Fig. 4A). Similarly, in assessing the sensitivity of paired mother and infant viruses to V3 glycan-targeting bNAb PGT128, for mother-infant pair 9105, all of the nontransmitted maternal viruses were uniformly neutralization sensitive to PGT128, whereas the infant-T/F virus was neutralization resistant to PGT128 (Fig. 4A), suggesting that the maternal-plasma N332 glycan-targeting bNAbs likely selected for this neutralization-resistant infant-T/F virus.

We next asked whether infant-T/F viruses 0616 and 9112, which appeared to be bNAb escape variants, were uniformly neutralization resistant to additional V2 glycan-targeting and V3 glycan-targeting bNAbs, respectively. Consistently with their PG9 neutralization sensitivities, infant-T/F virus 0616 and the closest maternal variant were also neutralization resistant to PG16 and PGT145, whereas the more genetically distant maternal variants were neutralization sensitive to these bNAbs (Fig. 4B). In observing the viral sequence in this region, V2 glycan neutralization-resistant infant-T/F virus 0616 and the closest 0601 maternal variant had large deletions within the V1V2 loop, whereas the nontransmitted neutralization-sensitive maternal variants did not have the V1V2 loop deletions (Fig. S7A and D), suggesting that these V1V2 deletions modulate neutralization resistance to V2 glycan-targeting bNAbs. However, as transmitted variants tend to have shorter V1V2 loops, these deletions may confer a general transmission fitness (50). Surprisingly, we did not observe plasma bNAb responses in transmitting HIV-infected woman 0601, suggesting that infant-T/F virus 0616 was selected in the absence of detectable V2 glycan-targeting bNAbs in plasma as measured by the differential neutralization activities against a panel of wild-type viruses and N160K and N160A mutant viruses that knock out PG9-like bNAb neutralizing activity (Fig. S7B). Moreover, we did not observe PG9-blocking activity in woman 0601 plasma, as measured by a blocking enzyme-linked immunosorbent assay (ELISA) (Fig. S7C). However, it is possible that HIV-infected woman 0601 had PG9-like neutralizing activity in her plasma that may have been too low to detect but that still applied immune pressure that led to escape mutations in circulating autologous maternal variants (51). Similarly, infant-T/F virus 9112 was neutralization resistant to N332 V3 glycan-targeting bNAbs PGT121, DH270, and 10-1074, whereas all of the nontransmitted maternal autologous viruses were uniformly neutralization sensitive to PGT121, 10-1074, and DH270 (Fig. 4C). Moreover, in mapping the specificity of the bNAb response in transmitting HIV-infected woman 9105, a V3 glycan-targeting plasma bNAb response was demonstrated (Fig. 3B and C), suggesting that the maternal bNAb responses shaped the selection of this neutralization-resistant infant-T/F virus.

10.1128/mBio.00176-20.7 FIG S7 Env V1V2 genetic signatures in amplicons of mother-infant pair 0601. (A) Amino acid residue deletions and mutations within the V1V2 loop in infant-T/F virus and the closest nontransmitted maternal *env* variant that are differentially neutralization sensitive to V2 glycan-targeting bNAbs. (B) Mapping of maternal-plasma PG9-like bNAb activity in transmitting HIV-infected woman 0601 against mutant viruses that ablate PG9-like bNAb activity. (C) The PG9-blocking activity of PG9-IgG1 MAb (positive control), a seronegative sample, and transmitting HIV-infected woman 0601. (D) Structural modeling of the molecular interactions of PG9 and differentially neutralization-sensitive maternal and infant-T/F viruses. Download FIG S7, PDF file, 0.2 MB. Copyright © 2020 Martinez et al. 2020 Martinez et al. This content is distributed under the terms of the Creative Commons Attribution 4.0 International license.

10.1128/mBio.00176-20.8 FIG S8 Signature sequence analysis of PG9 neutralization-resistant and -sensitive variants. (A) Amino acid residue at position 732 in uniformly neutralization-resistant woman 9105 nontransmitted maternal variants. (B) Amino acid residue positions in woman 0155 nontransmitted maternal variants that were differentially neutralization resistant to PG9. Download FIG S8, PDF file, 0.01 MB. Copyright © 2020 Martinez et al. 2020 Martinez et al. This content is distributed under the terms of the Creative Commons Attribution 4.0 International license.

### Molecular determinants of V3 glycan bNAb-mediated neutralization resistance of infant-T/F virus.

**FIG 5:**
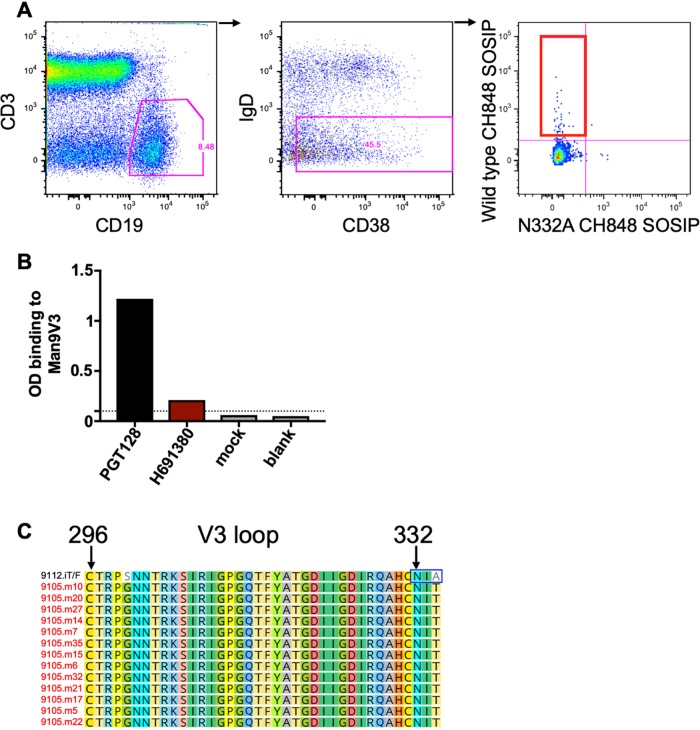
Maternal N332 glycan-targeting memory B cells and infant-T/F virus V3 glycan genetic signatures in mother-infant pair 9105. (A) N332 V3 glycan-specific peripheral blood memory B cells as defined by CD19^+^ IgD^–^ and CD38 all are shown by the red gate. (B) OD binding of N332 glycan-targeting monoclonal antibody H691380 isolated from woman 9105 against the Man_9_-V3 peptide. The dashed lined denotes the positivity cutoff. (C) V3 loop amino acid residues in woman 9105 nontransmitted maternal Env variants and infant-T/F virus 9112. Maternal Env amplicons from transmitting women are shown in the red font, and the infant-T/F virus is in the black font.

As we measured maternal-plasma N332 V3 glycan-targeting plasma bNAbs in transmitting HIV-infected woman 9105, who transmitted a V3 glycan bNAb-resistant variant to her infant (Fig. 3A), we sought to determine whether this transmitting woman had detectable N332 V3 glycan-targeting memory B cells in her peripheral blood. We sorted memory B cells, as defined by CD19^+^ IgD^–^ CD38, all from PBMCs, and examined the frequency of these cells that differentially bound to a HIV clade C wild-type CH848 trimeric mutant, which has a disulfide between residues 501 and 605 (SOS) and Ile to Pro (IP) mutation at residue 559, which are together designated SOSIP, but did not bind the N332A mutant CH848 trimeric SOSIP (Fig. 5A). Interestingly, transmitting woman 9105 had 44 out of 3,517 (1.2%) memory B cells that selectively bound to the wild-type CH848 SOSIP but not to the N332A mutant CH848 SOSIP. In fact, a monoclonal antibody isolated from these memory B cells (H691380) bound a Man_9_-V3 glycopeptide which has previously been shown to recapitulate the Env epitope of N332 V3 glycan-targeting bNAbs (38, 52) but did not bind to the V3 deglycosylated control peptide (Fig. 5B; Table S2), suggesting that this transmitting woman had N332 V3 glycan-targeting memory B cells in the periphery that gave rise to the plasma N332 V3 glycan-targeting bNAbs (Fig. 3A).

We then assessed the V3 loop amino acid sequence of the V3 glycan-targeting bNAb infant-T/F virus to determine whether these maternal-plasma N332 V3 glycan-targeting bNAbs selected for mutations that ablate the N332 V3 glycan signature in the 9112 infant-T/F virus. Notably, N332 V3 glycan neutralization-resistant infant-T/F virus 9112 had a T334A mutation, which ablated the N332 glycosylation signature that is required for the glycan attachment at this site, whereas all of the neutralization-sensitive paired autologous maternal viruses had an intact N332 glycosylation signature (Fig. 5C). Taken together, the measurement of maternal-plasma N332 V3 glycan-targeting responses in transmitting woman 9105 (Fig. 3B and C), the presence maternal peripheral blood memory B cells that bind to the N332 glycan-containing CH848 trimeric SOSIP (Fig. 5A), and the N332 glycosylation signature that is present in all of the sampled maternal 9105 viruses yet is markedly absent in the paired infant-T/F virus (Fig. 5C) suggest that maternal-plasma N332 V3 glycan-targeting bNAbs drove the selection of the N332 V3 glycan neutralization-resistant virus as the infant-transmitted variant.

## DISCUSSION

The development of a safe and effective maternal HIV vaccine will require the characterization of maternal humoral responses that are capable of blocking infant HIV infection. We previously demonstrated that V3-targeted antibodies neutralize tier 2 autologous HIV-1 strains and that this activity constrained the native Env trimer *in vivo* to a neutralization-resistant phenotype (22). Moreover, we previously defined maternal humoral correlates of protection against MTCT of HIV and demonstrated that maternal linear V3 epitope-specific IgG binding and tier 1 virus-neutralizing responses were associated with a reduced risk of MTCT (17). In a follow-up study, we showed that the same maternal V3-specific IgG responses targeted the C-terminal region of the V3 loop (43). The mapping of maternal V3-specific binding and neutralizing responses to the same region within the V3 loop led us to hypothesize that the potential mechanism of these protective responses was mediated through autologous-virus neutralization by weakly neutralizing antibodies directed against the linear portion of the V3 loop. To directly test that hypothesis, we obtained maternal Env-specific IgG MAbs from nontransmitting and transmitting HIV-infected U.S. and Malawian women and tested their neutralization activities against maternal circulating viruses present near the time of infant exposure/infection (nontransmitted variants) and against infant-T/F viruses. Previous studies determined that MTCT of HIV involves a genetic bottleneck from which only one or a few viruses are transmitted from a genetically diverse maternal virus population (53, 54). Accordingly, transmitting HIV-infected women in this study had considerable plasma *env* genetic diversity (see Fig. S1 in the supplemental material), and the selection of one or a few infant-T/F viruses from the maternal virus population was observed (Table S1 and Fig. S1) (25, 53). It is interesting that *in utero*-transmitting woman 3902 had at least two major plasma viral lineages and that her paired infant was infected with at least three distinct infant-T/F viruses (Table S1 and Fig. S1). In contrast, HIV-infected women 155.1, 0601, and 9105 each had two major plasma viral lineages, and their paired infants were infected with only one infant-T/F virus. However, it is unclear from this limited number of mother-infant pairs whether maternal-plasma *env* diversity is directly related to the number of infant-T/F viruses that initiate infection, and this observation should be followed up in larger cohorts.

Previous studies assessed maternal-plasma Env-specific IgG binding and neutralizing responses and their association with MTCT risk (17, 25, 43). Until now, no study has examined the maternal Env-specific B cell repertoire at the monoclonal antibody level in the context of MTCT of HIV. From our B cell repertoire analysis in nontransmitting and transmitting HIV-infected women, we found similar frequencies of V1V2, V3, and CD4bs-specific IgG MAb responses in nontransmitting and transmitting women (Table S2), suggesting that there is no difference in the epitope specificity distributions in the nontransmitting versus transmitting mothers included in this study. While we did not find differences between the V_H_ gene usage or somatic mutation levels of Env-specific IgG MAbs obtained from transmitting mothers and those of nontransmitting mothers (Fig. 1A and B), we detected higher somatic mutation levels of maternal V1V2-specific IgG responses in both nontransmitting and transmitting mothers than in gp120, V3, and CD4 binding site-specific IgG responses in HIV-infected mothers (Fig. 1D). However, the higher levels of somatic mutations in V1V2-specific IgG MAbs than in gp120, V3, and CD4 binding site-specific IgG MAbs did not lead to overall higher levels of autologous-virus-neutralizing activity in either nontransmitting or transmitting mothers (Fig. S3 and S4). It is not entirely clear why V1V2-specific IgG MAbs had higher levels of somatic cells mutated in this small group of HIV-infected mothers, but a potential explanation is that the V1V2 loop may be more immunogenic than other epitopes and in turn may select for more highly evolved V1V2-specific IgG responses. We did not detect any maternal autologous-virus neutralization activity by gp120, V1V2, or CD4 binding site-specific IgG MAbs. A potential reason that explains the lack of concordance in the neutralization activity of maternal plasma and the paired gp120, V1V2, and CD4 binding site-specific IgG MAbs is that the consensus MconSgp120 protein used in antigen-specific B cell sorting was limited in its ability to isolate B cells producing autologous-virus-neutralizing antibodies targeting autologous envelopes of these specificities. Moreover, two additional limitations that may explain the lack of agreement of the autologous-virus neutralization activity in plasma and MAbs is that (i) mother-infant pairs were selected based on adequate maternal PBMC availability near the time of delivery and not autologous-virus plasma neutralization activity and (ii) maternal and paired infant viruses were not always from the identical time points due to limitations in sample availability.

Maternal linear V3 epitope-specific IgG binding responses have been associated with a reduced risk of MTCT (17). Moreover, linear V3 epitope-specific IgG can neutralize HIV isolates from distinct clades and different neutralization resistance categories, including tier 1A, tier 1B, and tier 2 viruses (22, 55, 56). Vaccine-elicited V3-specific IgG responses in the moderately efficacious RV144 vaccine trial were highly cross-reactive with V3 cyclic peptides from diverse clades (57). A viral sieve analysis comparing the breakthrough viruses isolated from RV144 vaccinees to viruses isolated from placebo control individuals revealed that N-terminal amino acid residue 307 and C-terminal amino acid residue 317 modulated vaccine efficacy (57). Similarly, C-terminal amino acid residues within the V3 loop were found to be important for the binding and neutralization activity of maternal V3-specific IgG responses that were associated with a reduced MTCT risk (43). Here, we found that out of 17 total linear V3 epitope-specific monoclonal antibodies, 7 (41%) weakly neutralized 3 of 72 (4%) maternal autologous viruses in both nontransmitting and transmitting women. Interestingly, these weakly neutralizing linear V3 epitope-specific IgG MAbs similarly target amino acid residues at the N and C regions of the V3 crown (Fig. S5). While the MconSgp120 protein bait is a group M consensus Env that was specifically designed to capture the breadth of clade B and clade C Env diversity, it is possible that this Env bait biases for selecting heterologous- and not autologous-virus-neutralizing antibodies, potentially explaining the lack of autologous-virus neutralization activity by gp120, V1V2, and CD4 binding-specific IgG. Yet despite the limited overall autologous-virus neutralization activity, maternal linear V3 epitope-specific IgG MAbs had measurable, albeit low, levels of autologous-virus neutralization activity in this small cohort. These findings provide a proof of concept that V3-specific IgG MAbs can neutralize a small proportion of nontransmitted maternal viruses and that their binding activity depends on the same N- and C-terminal regions that are important for binding and neutralization for protective maternal-plasma linear V3 epitope-specific IgG responses (43). An interesting observation is that maternal variants that were neutralization sensitive to linear V3 epitope-specific IgG MAbs were not neutralization sensitive to other specificities (i.e., CD4 binding site-specific IgG). However, speculatively, it is possible that the Env-specific IgG MAbs isolated in our study have different recognition sensitivities of autologous viruses, as was reported for linear V3 and CD4bs-specific IgG responses in chronically infected individuals (22). Another potential explanation is that the maternal variants that are neutralization sensitive to linear V3-specific IgG MAbs have a more “open” gp120 envelope, potentially explaining their susceptibility to these weakly neutralizing antibodies (56). Moreover, in a previous study by our group, we reported that infant-T/F viruses were overall more neutralization resistant to maternal autologous plasma than nontransmitted maternal variants were (25). Yet, in this study, this was not the overall observed trend. Potential reasons for these differences are likely the smaller number of mother-infant pairs in this study than in that of Kumar et al. (25) and, thus, its lower power. Moreover, Kumar et al. focused exclusively on peripartum transmission cases, whereas this study included both peripartum and *in utero* transmissions.

While maternal-plasma Env-specific IgG neutralizing antibody responses with limited neutralization breadth were shown to be associated with reduced MTCT risk (17), it remains unclear if maternal-plasma broadly neutralizing antibody responses are protective or a deleterious risk factor for MTCT of HIV. Ghulam-Smith et al. recently reported that transmitting clade C HIV-infected women had more broad neutralizing responses than nontransmitting women (19). We similarly detected maternal-plasma bNAb responses in two of four transmitting HIV-infected women and in one of three nontransmitting women (Fig. 3), further demonstrating that plasma bNAb activity can be present in both nontransmitting and transmitting women. Yet, we demonstrated evidence of neutralization escape in infant-T/F virus 9112 via a specific T334A mutation that ablates the N332 glycosylation site, which leads to escape from V3 glycan-targeting bNAbs. This finding suggests that infant-T/F virus 9112 is likely a variant that escaped maternal-plasma N332 V3 glycan-targeting bNAb responses, which were also identified in the memory B cell repertoire (Fig. 5A and B; Table S2). Indeed, infant-T/F virus 9112 was broadly neutralization resistant to V3 glycan-targeting bNAbs PGT128, PGT121, 10-1074, and DH270. While infant-T/F virus 9112 was more sensitive to maternal plasma than the nontransmitted maternal variants, these findings clearly demonstrate that a single bNAb lineage (i.e., the V3 glycan-targeting bNAb lineage) present in maternal plasma is not sufficient for protection against MTCT, in particular against variants that have escaped bNAb activity. The neutralization resistance of infant-T/F virus 9112 to V3 glycan-targeting bNAbs despite its neutralization sensitivity to paired maternal plasma is likely explained by other polyclonal neutralizing antibody responses present in 9105 plasma (Fig. 3B; Fig. S4). We also detected maternal Env variants that were neutralization resistant to V2 and V3 glycan-targeting bNAbs in the absence of plasma bNAb responses in these women, and these neutralization-resistant variants were not vertically transmitted (Fig. 4A). Thus, it is likely that additional selection factors beyond maternal-plasma bNAb responses account for the transmission of bNAb-resistant variants. For example, it is possible that nonneutralizing functions, such as antibody-dependent cellular cytoxicity (ADCC), also shape the selection of infant-T/F viruses. Nevertheless, the presence of maternal-plasma bNAbs may select for bNAb escape variants with increased replication fitness in both the mother with plasma bNAb activity and the passively immunized fetus with maternal IgG and that this may be a mechanism of the selection of infant-T/F variants.

While we observed low levels of autologous-virus neutralization by V3-specific IgG MAbs in nontransmitting and transmitting mothers, our data agree with previous studies in that maternal linear V3 epitope-specific IgG responses can neutralize nontransmitted circulating autologous viruses in plasma (17, 43). This study is also the first to report an N332 V3 glycan neutralization-resistant infant-T/F virus in association with maternal-plasma N332 V3 glycan-targeting bNAbs (Fig. 3 and 5). Collectively, these findings suggest that maternal-plasma HIV variants that initiate infant infections may be selected by escape from maternal-plasma broadly neutralizing activity. The finding that maternal-plasma bNAbs can select neutralization-resistant infant-T/F viruses should be considered in the safety of HIV vaccine trials that test the induction of bNAbs to prevent transmission, especially in pregnant HIV-infected women. Moreover, these data should also be considered in ongoing clinical trials testing passively administered bNAbs as suppressive antibody therapies, particularly in pregnant HIV-infected women, as they suggest that a single bNAb specificity can select for neutralization-resistant infant-T/F viruses that will be resistant to this class of potential bNAb-based therapeutics.

## MATERIALS AND METHODS

### Study design.

Four transmitting mother-infant pairs from the North American Women Infant Transmission Study (WITS) cohort and the Malawian Center for HIV/AIDS Vaccine Immunology 009 (CHAVI009) cohort were selected based on the following criteria: the infants had to have been HIV infected *in utero* or peripartum and had an identifiable infant-T/F virus in their plasma, as determined by single-genome analysis (SGA), and paired maternal-plasma samples had to have been obtained within 4 months of the transmission of the infant-T/F viruses. Mother infant pairs in this study were not selected based on high levels of maternal-plasma autologous-virus-neutralizing activity but instead were selected on the availability of maternal PBMCs near the time of infant infection (see Table S1 in the supplemental material). As expected, pregnant U.S. women from the WITS cohort were infected primarily with clade B HIV, and pregnant Malawian women from the CHAVI009 cohort were infected primarily with clade C HIV (49). Both cohorts and their enrollment criteria have been previously described (17, 49, 58). *In utero* transmission was defined by a positive PCR result at delivery. Peripartum transmission was defined by a negative HIV PCR or culture from peripheral blood samples collected within 7 days of birth, with a subsequent positive result >7 days after birth. Three nontransmitting women were also selected from the WITS and CHAVI009 cohorts. Clinical characteristics of the maternal and infant pairs used in this study are in Table S1. Maternal and infant patients that were used to isolate single-genome amplicons and M.ConSgp120-specific memory B cells are listed in Table S1.

The study CHAVI009 was approved by the College of Medicine Research and Ethics Committee in Malawi and by all participating institutions’ institutional review boards (IRBs). The WITS study was approved by the IRB of each study site. The study sites include Massachusetts (subsites in Boston and Worcester), New York (centers in Manhattan and Brooklyn), Texas (Houston), Puerto Rico (San Juan), and Illinois (Chicago). After the original WITS study protocol was approved, written informed consent was obtained from all participants (59). Maternal and infant samples from the WITS and CHAVI009 cohorts were received as deidentified material and were deemed “not human subject research” by the Duke University School of Medicine IRB.

### HIV e*nv* SGA isolation.

Viral RNA (vRNA) was isolated from maternal and infant plasma using EZ1 Virus minikit 2.0 on BioRobot EZ1 (Qiagen) according to manufacturer specifications. cDNA was reverse transcribed with 1× reaction buffer, 0.5 mM each deoxynucleoside triphosphate (dNTP), 5 mM dithiothreitol (DTT), 2 U/ml RNaseOUT, 10 U/ml of SuperScript III reverse transcription mix (Invitrogen), and 0.25 mM antisense primer 1.R3.B3R (5′ACTACTTGAAGCACTCAAGGCAAGCTTTATTG-3′) using 20 μl of vRNA. The cDNA was used for first-round PCR amplification using Platinum *Taq* high-fidelity DNA polymerase (Invitrogen) by endpoint dilution PCR in 96-well plates (Applied Biosystems, Inc.). Only PCRs in which <30% of the reactions were positive were used in order to maximize the likelihood of isolating single-genome amplicons. First-round PCR HIV *env* single amplicons were generated using the 07For7 (5′-AAATTAYAAAAATTCAAAATTTTCGGGTTTATTACAG-3′) and 2.R3.B6R (5′-TGAAGCACTCAAGGCAAGCTTTATTGAGGC-3′) primer pairs. Second-round PCR amplification was performed with primers VIF1 (5′-GGGTTTATTACAGGGACAGCAGAG-3′) (nucleotides [nt] 5960 to 5983 in the HXB2 *tat* coding region) and Low2c (5′-TGAGGCTTAAGCAGTGGGTTCC-3′) (nt 9413 to 9436 in HXB2 *nef*) and with 2 μl of the first-round PCR mixture as the template. All first-round PCRs were performed in 20-μl reaction mixtures using 1× buffer, 2 mM MgSO_4_, 0.2 mM each dNTP, 0.2 μM each primer, and 0.025 U/μl Platinum *Taq* high-fidelity polymerase (Invitrogen). First-round PCR amplification conditions were 1 cycle of 94°C for 2 min and 35 cycles of 94°C for 15 s, 58°C for 30 s, and 68°C for 4 min, followed by 1 cycle of 68°C for 10 min. Second-round PCR conditions were 1 cycle of 94°C for 2 min and 45 cycles of 94°C for 15 s, 58°C for 30 s, and 68°C for 4 min, followed by 1 cycle of 68°C for 10 min. Second-round PCR amplicon products were visualized by agarose gel electrophoresis, and positive products were sequenced using an ABI3730xl genetic analyzer (Applied Biosystems). The final 3′-half genome PCR product was ∼4,160 nucleotides in length and included *env* gp160. Partially overlapping *env* sequences from each PCR amplicon were assembled and edited using Sequencher (Gene Codes, Inc.). Sequences with double peaks per base read were discarded. HIV *env* sequence alignments and phylogenetic trees were generated using ClustalW, and Highlighter plots were created using Los Alamos National Laboratory (LANL) HIV tools at https://www.hiv.lanl.gov/content/sequence/HIGHLIGHT/highlighter_top.html.

### Identification of infant-T/F HIV *env* sequences.

All maternal and infant envelope sequences were aligned using the Gene Cutter tool available at the LANL website (http://www.hiv.lanl.gov/content/sequence/GENE_CUTTER/cutter.html) and edited manually. Full-length HIV envelope sequences were manually trimmed using Seaview (60). The infant-T/F *env* virus sequences were visually examined in phylogenetic trees and Highlighter plots. Infant consensus sequences of the major T/F lineage were created using the LANL Consensus Maker tool (http://www.hiv.lanl.gov/content/sequence/CONSENSUS/consensus.html). Highlighter plots and phylogenetic trees were rooted on the midpoint of the major *env* variant in infants infected with one or more T/F viruses. Maternal and infant envelope sequences were aligned using Bio-NJ phylogeny (Mega 6 Software). The number of infant-T/F viruses in each patient was determined by visual inspection of the phylogenetic tree and Highlighter plotting of infant-maternal *env* sequence alignments. *env* hypermutation was also evaluated using the tool Hypermut (http://www.hiv.lanl.gov/content/sequence/HYPERMUT/hypermut.html). *env* sequences with significant enrichment for hypermutation (*P* < 0.1) were removed from the alignment and not included in further analysis. When an *env* sequence had high hypermutation frequencies (61), positions within the APOBEC signature context were removed. The timing of infection was inferred using Poisson-Fitter (http://www.hiv.lanl.gov/content/sequence/POISSON_FITTER/pfitter.html), based on the accumulation of random mutations from the most recent common ancestor (MRCA), after removal of putative recombinants and/or positions in the alignment that are in a hypermutated context for all 4 infant infections (42). For infants infected with 2 or more T/F viruses, only the major *env* variant was analyzed to obtain the time since the infection. The defined *env* mutation rate was 2.16 × 10^−5^ per base per generation. Values were reported in numbers of days, with a 95% confidence interval and a goodness-of-fit *P* value. As we amplified >20 *env* sequences per infant, we had a >90% confidence that any *env* variants within the population with a frequency of 0.5% or higher were sampled. All of the infant-T/F viruses fit a Poisson distribution.

### Selection of maternal *env* sequences.

To select nontransmitted maternal variants and capture the most divergent sequences from the infant-T/F viruses, we devised an algorithm in R as follows. We first identify the most variable positions in the amino acid alignment and rank of all sequences with respect to the frequencies at these positions. Sequences are then selected starting from the most divergent based on motif coverage as observed in the alignment and in the phylogenetic tree (in other words, if a group of diverging sequences all share the same motif, only one in the group and/or tree node is selected), as described previously (25).

### Cloning of maternal and infant-T/F *env* SGAs.

A 5′-CACC end was introduced using primers Env1A (5′-CACCTTAGGCATCTCCTATGGCAGGAAGAAG-3′) and Rev19 (5′-ACTTTTTGACCACTTGCCACCCAT-3′) into maternal and infant-T/F virus amplicons in the first-round PCR. Maternal and infant PCR products were ligated into pcDNA3.1 directional TOPO vectors (Invitrogen). Phusion high-fidelity PCR master mix with high-fidelity (HF) buffer was used according to the manufacturer guidelines (New England BioLabs). *env* cloned-gene plasmids were then transformed into XL10 gold chemically competent Escherichia coli cells. Transformed bacterial cultures were grown at 37°C for 16 h on LB-ampicillin plates, and single colonies were selected. Bacterial colonies were propagated in liquid broth containing 100 μg/ml ampicillin, and plasmids were miniprepped and quality controlled by BamHI and XhoI (New England BioLabs) restriction enzyme digestion. Plasmids containing the *env* insert of the correct size were sequenced to confirm 100% sequence identity with the original *env* infant consensus sequence. Plasmids were propagated on a large scale, megaprepped using a commercially available kit (Zymo Research), and resequenced to confirm 100% sequence identity.

### HIV pseudovirus preparation in 293T cells.

HIV *env* pseudoviruses were prepared by transfecting 60%-confluent 293T cells (ATCC, Manassas, VA) with 4 μg of *env* plasmid DNA and 8 μg of *env-*deficient HIV plasmid DNA using the FuGene 6 transfection reagent (Roche Diagnostics) as described previously (62). Two days after transfection, the culture supernatant containing pseudoviruses was harvested, filtered using 0.22-μm sterile filters, aliquoted, and stored at –80°C. An aliquot of freeze-thawed pseudovirus was used to measure pseudovirus infectivity in TZM-bl cells. Twenty microliters of pseudovirus was distributed in duplicate to 96-well flat-bottom plates (Co-star), 10,000 TZM-bl cells were grown in media (Dulbecco’s modified Eagle’s medium [DMEM]–10% fetal bovine serum [FBS] containing HEPES), and 10 μg/ml of DEAE-dextran was added to each well. After 48 h of incubation at 37°C in 5% CO_2_, 100 μl of medium was removed from pseudovirus-containing wells and 100 μl of luciferase reagent Bright-Glo was added; the plates were then incubated at room temperature (RT) for 2 min. One hundred microliters of the cell lysate was transferred to a 96-well solid-black plate (Costar), and the luminescence was read using a Victor plate reader (Perkin-Elmer).

### Isolation of Env-specific B cells from nontransmitting and transmitting HIV-infected women.

Peripheral Env-specific IgG-expressing memory B cells were obtained from 4 transmitting HIV-infected women and 3 nontransmitting HIV-infected women as described previously (17). Thawed PBMCs were stained with a cell viability marker (Life Technologies; Aqua Vital dye L34957), SAV-AF647 (Life Technologies; S21374), SAV-BV421 (BioLegend; 405225)-labeled M.ConSgp120, and the following antibodies: CD27 phycoerythrin (PE)-Cy7 (eBioscience), anti-IgG fluorescein isothiocyanate (FITC) (Jackson Immuno Research), IgD PE, CD19 allophycocyanin (APC)-Cy7, CD3 PE-Cy5, CD235a PE-Cy5 (BD Pharmingen), CD14 BV605, CD16 BV570 (Sony/iCyt), CD10 ECD, and CD38 APC AF700 (Beckman Coulter). Total B cells were gated as viable (Aqua Vital dye negative) CD14/CD16 and CD3/CD235a negative, and CD19 positive. HIV Env-specific, IgG-expressing memory B cells were further selected by gating for IgD-negative and M.ConSgp120 double-positive cells using dual-color (SAV-AF647 and SAV-BV421) antigen-specific labeling. To isolate maternal N332 V3 glycan-targeting memory B cells, we utilized the same sort strategy, except at the final sort gate, in which cells were selected based on differential binding to N332 glycan-containing CH848 trimeric mutant SOSIP-SAV-BV421 and N332A CH848 trimeric mutant SOSIP-SAV-AF647. Flow cytometric data were acquired on a BD Aria II fluorescence-activated cell sorter (FACS) (BD Biosciences) at the time of sorting, and the data were analyzed using FlowJo (Tree Star Inc.). Single-cell sorting of double MConSgp120-specific B cells was performed using a FACSAria II (BD Biosciences) to sort single cells into 96-well plates preloaded with an RNA stabilization cocktail as described previously (17).

### Variable heavy- and light-chain gene amplification, transient transfection, and screening ELISA.

B cell cDNA was generated by reverse transcription using random-hexamer primers and SuperScript III reverse transcriptase. B cell immunoglobulin variable heavy- and light-chain genes were amplified by nested PCR using forward and reverse primer sets as previously described (58). The IgG isotype was determined by sequence homology to germ line genes. Somatic hypermutation and inferred V(D)J rearrangement was determined by somatic-diversification analysis (SoDA) as previously described (63). Overlapping PCR was used to construct variable gene PCR products with full-length IgG1 (for heavy-chain) and kappa or lambda (for light-chain) cassettes for expression in 293T cells (64). The supernatants of transiently transfected 293T cells were screened for binding against a panel of HIV proteins and peptides by ELISA. Binding ELISAs were performed by coating highly binding 384-well plates (Corning) for 1 h at RT with M.ConSgp120, Con6gp120, MNgp120gDneg11, gp70MV3, V3.B (NNTRKSIHIGPGRAFYATGDIIGDIRQAHC), V3.C (KKKNNTRKSIRIGPGQTFYATGDIIGDIRQAHC), gp70BcaseAV1V2, Bio-V2.B (Bio-KKKTSIRDKVQKEYALFYKLDVVP), Bio-V2.1086C (Bio-KKKTELKDKKHKVHALFYKLDVVP), Bio-RV144.C5.2B (Bio-KKKSELYKYKVVKIEPLGVAPTKAKRRVVQREKRAV), YU2 core protein, YU2 core D368R mutant protein, MulVgp70, a scrambled peptide (HTGKYTYPTNIAIRGRGNKFRNKKI), a deglycosylated V3 control peptide, and a synthetic glycopeptide, Man_9_-V3, which recapitulates the Env epitope of V3 glycan-targeting bNAbs (52). Plates were washed once and blocked for 2 h at RT with SuperBlock (4% whey protein, 15% goat serum, and 0.5% Tween 20 diluted in 1× phosphate-buffered saline [PBS]). After a double wash, 10 μl of the transient-transfection supernatant was added in duplicate and incubated at RT for 1 h. Plates were washed twice, a horseradish peroxidase (HRP)-conjugated goat anti-human IgG antibody (Sigma-Aldrich) was used at a 1:10,000 dilution, and the plates were incubated at RT for 1 h. The plates were washed four times, and the SureBlue Reserve TMB substrate (KPL, Gaithersburg, MD) was added. Reactions were stopped with a stop solution (KLP, Gaithersburg, MD), and optical densities (ODs) were detected at 450 nm. Binding positivity cutoffs for each antigen were set as 3 times the background OD of the non-HIV-specific IgG MAb control transfection supernatant. MAbs were defined as gp120 specific if they bound to either MconSgp120, Con6gp120, or MNgp120gDneg11, as defined by a cutoff of 3 times the OD of the small-scale transfection non-HIV heavy- and light-chain positive-control supernatant. All gp120-specific MAbs were further categorized as V3 specific if they bound to gp70MNV3, V3.B, or V3.C; as V1V2 specific if they bound to gp70BcaseAV1V2, V2.B, or V2-1086C; and as CD4 binding site specific if they bound to the YU2 core but not the YU2 core D368R protein.

### Monoclonal antibody screening and large-scale production.

Twenty-seven of 242 MAbs obtained from three HIV-infected women and 30 out of 155 MAbs obtained from four transmitting HIV-infected women were selected for large-scale transfection based on their genetic diversity and strength of binding to an HIV Env antigen panel (Table S2). Heavy- and light-chain variable-gene-expressing plasmids encoding an IgG1 Fc region with S298A, E333A, K334A, and N434A mutations (GenScript) for optimized binding to FcγRIIIa were cotransfected using an ExpiFectamine 293 transfection kit in Expi293F (Thermo) cells at 2.5 million cells/ml in 100-ml flasks in suspension. Supernatants were harvested 5 days later, and IgG MAbs were purified using Pierce protein A beads (Thermo). MAbs were buffer exchanged with 60 ml of sterile 1× PBS. Purified IgG MAbs were tested at 5 μg/ml against the same HIV Env protein and peptide panel tested in the antibody-screening ELISAs to confirm Env binding specificity. Binding positivity cutoffs were set as twice the background OD of a CH65 (influenza virus-specific MAb) negative control for each antigen. Anti-HIV immunoglobulin (HIVIG) (NIH AIDS Reagent Program) was used as the positive control for each HIV antigen. Purified MAbs were tested by Western blotting and Coomassie blue staining to confirm MAb purity.

### V3-specific IgG MAb mapping ELISA.

V3 peptide-binding ELISAs were performed by coating highly binding 384-well plates (Corning) for 1 h at RT with V3.C (KKKNNTRKSIRIGPGQTFYATGDIIGDIRQAHC), V3.B (NNTRKSIHIGPGRAFYATGDIIGDIRQAHC), the V3.B K305Q I307T H308T mutant (NNTRQSTTIGPGRAFYATGDIIGDIRQAHC), the V3.B F317L A319T D322R mutant (NNTRKSIHIGPGRALYTTRIIGDIRQAHC), V3.B K305A (NNTRASIHIGPGRAFYATGDIIGDIRQAHC), V3.B I307A (NNTRKSAHIGPGRAFYATGDIIGDIRQAHC), V3.B H308A (NNTRKSIAIGPGRAFYATGDIIGDIRQAHC), V3.B F317A (NNTRKSIHIGPGRAAYATGDIIGDIRQAHC), V3.B A319K (NNTRKSIHIGPGRAFYKTGDIIGDIRQAHC), V3.B D322A (NNTRKSIHIGPGRAFYATGAIIGDIRQAHC), and V3.B F317A A319K D322A (NNTRKSIHIGPGRAAYKTGAIIGDIRQAHC). Plates were washed once and blocked for 2 h at RT with SuperBlock (4% whey protein, 15% goat serum, and 0.5% Tween 20 diluted in 1× PBS). After a double wash, V3-specific IgG MAbs at 5 μg/ml were serially diluted, added in duplicate, and incubated at RT for 1 h. Plates were washed twice, an HRP-conjugated goat anti-human IgG antibody (Sigma-Aldrich) was used at a 1:10,000 dilution, and the plates were incubated at RT for 1 h. The plates were washed four times, and the SureBlue Reserve TMB substrate (KPL, Gaithersburg, MD) was added. Reactions were stopped with a stop solution (KLP, Gaithersburg, MD), and ODs were detected at 450 nm. Binding positivity cutoffs for each V3 mutant peptide were defined as twice the background OD of the negative control, a flu-specific IgG CH65 MAb.

### Measurement of PG9 blocking activity by ELISA.

PG9 blocking activity ELISAs were done by coating high-binding 384-well plates (Corning) for 1 h at RT with 45 ng of ConCgp120. Plates were washed once and blocked with SuperBlock at RT for 1 h. A PG9-IgG1 positive-control MAb and VRC01-IgG1 negative-control MAb were diluted to 4 μg/ml, and an HIV-seronegative serum sample and HIV-infected woman 0601 plasma sample were diluted at a 1:10 dilution. Diluted MAb and plasma samples were added to the plates and were incubated for 1 h at RT. Plates were washed twice, and a biotinylated PG9-IgG1 MAb was added at a 2-fold serial dilution, starting at 4 μg/ml, and incubated for 1 h. Plates were washed twice, an HRP-streptavidin detector was added at a 1:10,000 dilution, and plates were incubated for 1 h. Plates were then washed four times and developed using a SureBlue Reserve TMB substrate. The substrate was neutralized by adding an equal volume of a stop solution (KLP, Gaithersburg, MD), and ODs were detected at 450 nm.

### HIV neutralization assays.

Serum/plasma samples were heat inactivated at 56°C for 1 h prior to being assayed. Murine leukemia virus SVA.MuLV was used as a negative control (65). Neutralizing-antibody activity was measured in 96-well culture plates by using Tat-regulated luciferase (Luc) reporter gene expression to quantify reduction in virus infection in TZM-bl cells (NIH AIDS Research and Reference Reagent Program). Assays were performed with HIV-1 *env*-pseudotyped viruses as described previously (66). We tested maternal Env-specific IgG MAbs against HIV tier 1 A isolates LS.SF162, MN.3, and MW965. To measure maternal-plasma bNAb activity, we tested viruses in the nine-virus global panel: 246F3, BJOX2000, CE1176, CNE55, TRO.11, X1632, CEO217, 25710, and CH119. To map the specificities of maternal-plasma bNAbs, we tested the following mutant viruses: BJOX2000 N160K, BJOX2000 N332A, TRO.11 N332A, TRO.11 N276Q, TRO.11 N279A, and TRO.11 G458Y. Test samples were diluted over a range of 1:20 to 1:43,740 in cell culture medium and preincubated with virus (∼150,000 relative light unit equivalents) for 1 to 1.5 h at 37°C. Ten thousand TZM-bl cells and 10 μg/ml of DEAE-dextran per well were added. After a 48-h incubation, cells were lysed and luminescence was measured using Bright-Glo in black 96-well plates using a Victor plate reader (Perkin-Elmer). Neutralization titers were defined as the sample dilution or monoclonal antibody concentration at which a 50% reduction of relative luminescence units (RLU) was observed in the virus control wells after subtraction of background RLU in cell control wells. A response was considered positive if the plasma ID_50_ against infant-T/F viruses was at least 3 times higher than the ID_50_ versus SVA.MLV. A flu virus-specific CH65 IgG MAb was included as a negative control. CD4bs-targeting VRC01 IgG, V2 glycan-targeting PG9 IgG, MPER-targeting DH512 IgG, and V3 glycan-targeting PGT128 IgG broadly neutralizing antibodies were included as positive controls.

### Statistical analysis.

The permutation test and the random-effect generalized linear model analysis (package lme4) were written and run in R (67; http://www.R-project.org/). *P* values of less than 0.05 were considered significant. Sites for potential genetic signatures were identified by testing each amino acid across Env alignments from all maternal sequences for associations with sensitivity to each bNAb using the LANL tool GenSig (47; https://www.hiv.lanl.gov/content/sequence/GENETICSIGNATURES/gs.html). In order for GenSig to perform the phylogenetically corrected Fisher tests, PG9 and VRC01 50% infective concentration (IC_50_) values were dichotomized as 1 if the value fell within the highest quartile and 0 otherwise. Given the limited sample size, we used a stringent criterion by reporting only sites that were significant after phylogenetic correction and that have previously been published to be associated with bNAb sensitivity or resistance in the literature.
