# Supplementary material for: Maternal Broadly Neutralizing Antibodies Can Select for Neutralization-Resistant, Infant-Transmitted/Founder HIV Variants
Source: mBio. 2020 Mar 10;11(2):e00176-20. doi: 10.1128/mBio.00176-20 (PMC7064758; doi:10.1128/mBio.00176-20)
Supplement: TABLE S1 [file mBio.00176-20-st001.pdf]

Table S1. Maternal clinical characteristics, number of amplified infant and maternal Env sequences, and timepoints of maternal and infant plasma and PBMC samples of U.S. and Malawian HIV-infected non-transmitting and transmitting women.

Transmission status	Maternal ID	Mode of transmission	Cohort	Mode of delivery	Maternal CD4+ T cell count (cells/mm3)	Maternal viral load (copies/ml)	Maternal SGA timepoint	Maternal PBMC sample	Paired Infant ID	Infant T/F SGA timepoint	Number of amplified infant Env gene SGAs	Estimated number of infant T/F viruses	Infant age at sample collection (days)	Estimated days since Most Recent Common Ancestor (MRCA) (95% CI)	Notes	Number of amplified maternal Env gene SGAs
Non-transmitter	193.1	NA[1]	WITS	UNK[2]	787	29,337	2 months PP[3]	6 months PP	NI[5]							
Non-transmitter	5807	NA	CHAVI009	vaginal	188	58,915	1 month PP	3 months PP	NI							
Non-transmitter	0301	NA	CHAVI009	vaginal	406	75,934	1 month PP	3 months PP	NI							
Transmitter	155.1	peripartum	WITS	c-section	318	87,193	delivery	2 months PP	155.11	4 months post infection	23	1	120 days	63 (50,76)	Fits a Poisson distribution	39
Transmitter	0601	peripartum	CHAVI009	vaginal	295	70,267	delivery	1 month PP	0616	1 month post infection	31	1	38 days	66 (50,82)	Fits a Poisson distribution	29
Transmitter	9105	*in utero*	CHAVI009	vaginal	266	5,207	delivery	2 months AP and 1 month PP	9112	delivery (cord blood)	20	1	delivery	18 (12,24)	Fits a Poisson distribution	26
Transmitter	3902	*in utero*	CHAVI009	vaginal	213	163,069	1 month AP[4]	3 months PP	3915	delivery (cord blood)	47	3	delivery	32 (21,43)	Fits a Poisson distribution	36

[1]NA = not applicable

[2]UNK = Unknown

[3]PP: Postpartum

[4]AP: Antepartum

[5]NI: Not included
