# Supplementary material for: Maternal Broadly Neutralizing Antibodies Can Select for Neutralization-Resistant, Infant-Transmitted/Founder HIV Variants
Source: mBio. 2020 Mar 10;11(2):e00176-20. doi: 10.1128/mBio.00176-20 (PMC7064758; doi:10.1128/mBio.00176-20)
Supplement: FIG S1 [file mBio.00176-20-sf001.pdf]

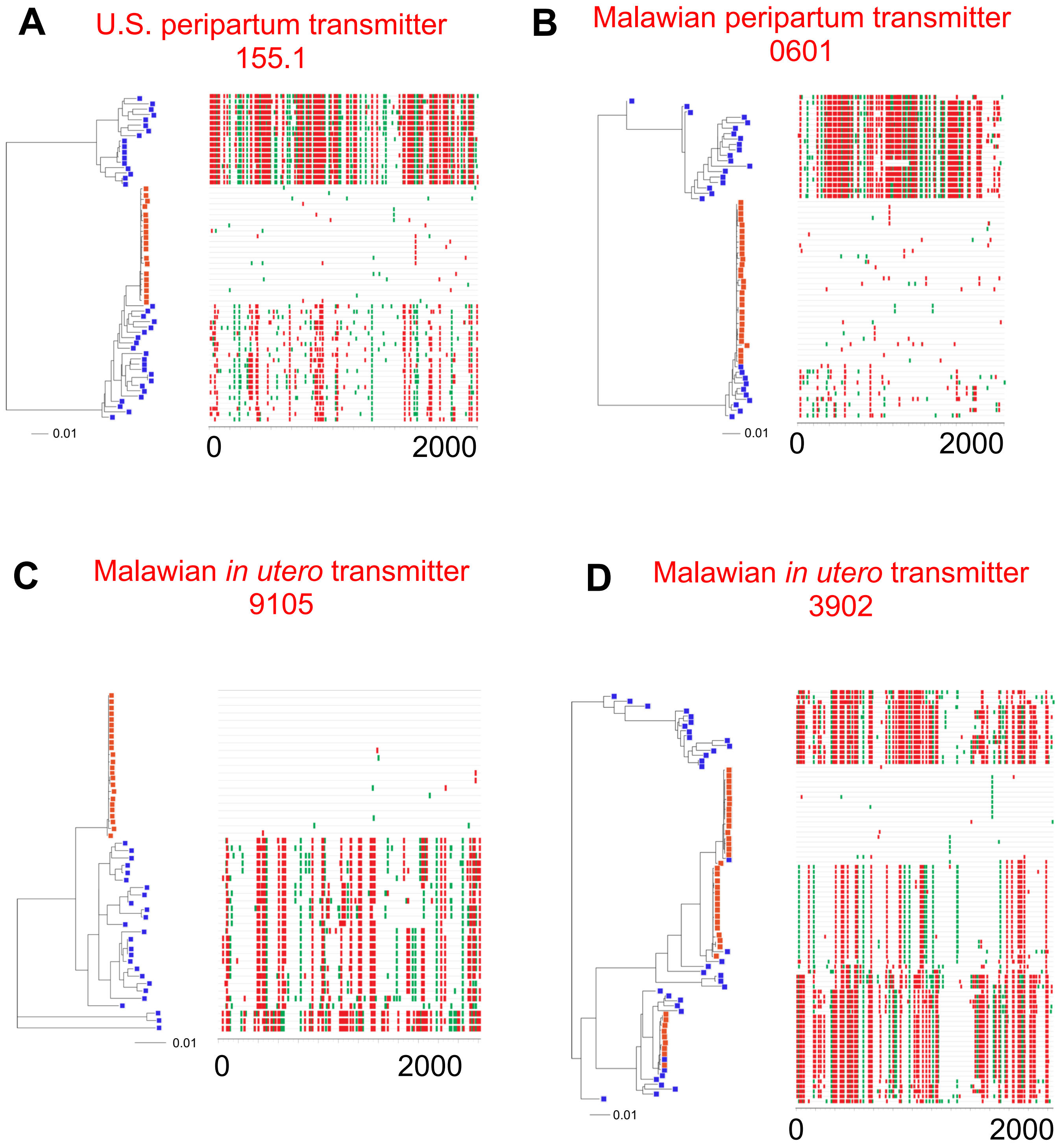
A
U.S. peripartum transmitter
155.1
0.01
0
2000
B
Malawian peripartum transmitter
0601
0.01
0
2000
C
Malawian in utero transmitter
9105
0.01
0
2000
D
Malawian in utero transmitter
3902
0.01
0
2000
