# Supplementary figures and images for: Maternal Broadly Neutralizing Antibodies Can Select for Neutralization-Resistant, Infant-Transmitted/Founder HIV Variants
Source: mBio. 2020 Mar 10;11(2):e00176-20. doi: 10.1128/mBio.00176-20 (PMC7064758; doi:10.1128/mBio.00176-20)

**A** U.S. non-transmitter  
193.1

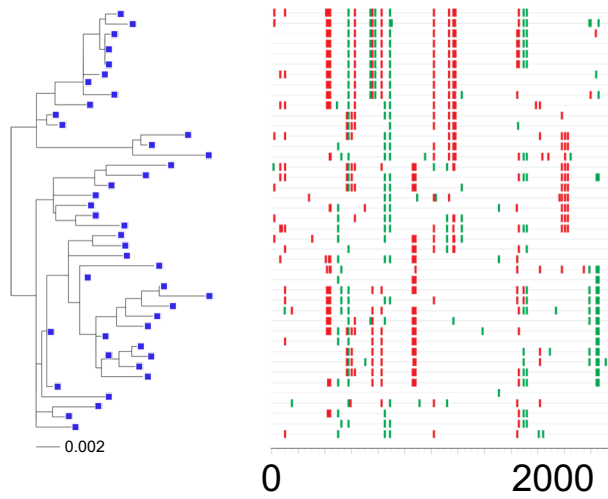

**B** Malawian non-transmitter  
5807

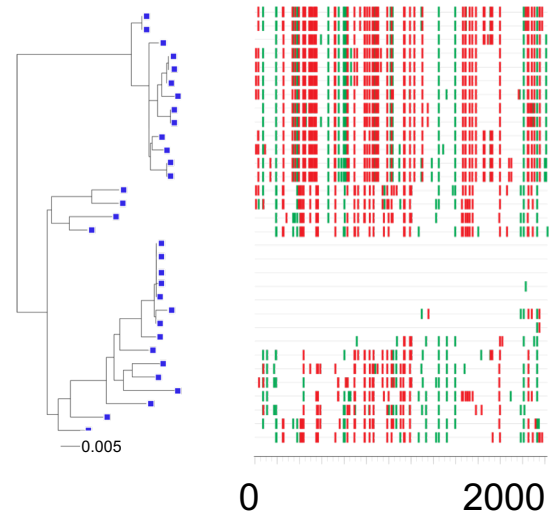

**C** Malawian non-transmitter  
0301

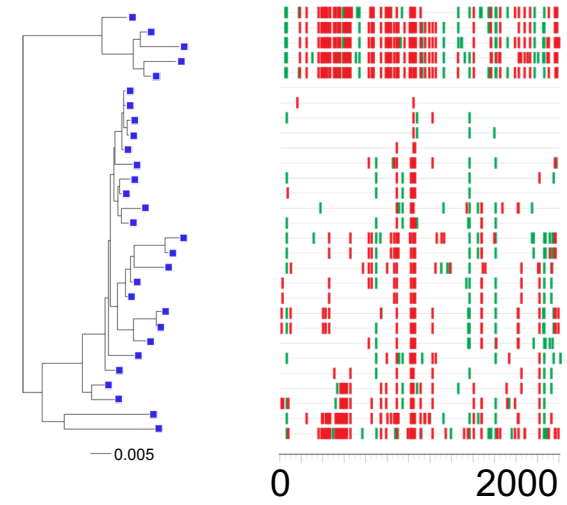

Supplement: FIG S2 [file mBio.00176-20-sf002.pdf]
