# Supplementary material for: Maternal Broadly Neutralizing Antibodies Can Select for Neutralization-Resistant, Infant-Transmitted/Founder HIV Variants
Source: mBio. 2020 Mar 10;11(2):e00176-20. doi: 10.1128/mBio.00176-20 (PMC7064758; doi:10.1128/mBio.00176-20)
Supplement: FIG S3 [file mBio.00176-20-sf003.pdf]

				maternal viruses								
	Specificity	Sample	SVA.MulV	193.1m3	193.1m10	193.1m18	193.1m29	193.1m37	193.1m38	193.1m43	193.1m46	193.1m47
193.1	polyclonal	plasma	<20	53	28	30	31	40	27	32	44	30
	CD4bs-specific	H650293	NT	>50	>50	>50	>50	>50	>50	>50	>50	>50
	CD4bs-specific	H650301	NT	>50	>50	>50	>50	>50	>50	>50	>50	>50
	CD4bs-specific	H650310	NT	>50	>50	>50	>50	>50	>50	>50	>50	>50
	V3-specific	H650322	NT	>50	>50	>50	>50	>50	>50	>50	>50	>50
	Gp120-specific	H650332	NT	>50	>50	>50	>50	>50	>50	>50	>50	>50
	CD4bs-specific	H650341	NT	>50	>50	>50	>50	>50	>50	>50	>50	>50
	Gp120-specific	H650342	NT	>50	>50	>50	>50	>50	>50	>50	>50	>50
	V3-specific	H650367	NT	>50	>50	>50	>50	>50	>50	>50	>50	>50

ID50	IC50 (μg/ml)
<20	>50
<100	21-49.9
>100	5-20.9

NT: not tested

				maternal viruses				
	Specificity	Sample	SVA.MulV	0301m14	0301m17	0301m18	0301m27	0301m29
0301	polyclonal	plasma	<20	62	<20	37	32	45
	CD4bs-specific	H650145	NT	>50	>50	>50	>50	>50
	V1V2-specific	H650146	NT	>50	>50	>50	>50	>50
	V1V2-specific	H650148	NT	>50	>50	>50	>50	>50
	V1V2-specific	H650154	NT	>50	>50	>50	>50	>50
	CD4bs-specific	H650181	NT	>50	>50	>50	>50	>50
	CD4bs-specific	H650182	NT	>50	>50	>50	>50	>50
	Gp120-specific	H650192	NT	>50	>50	>50	>50	>50
	Gp120-specific	H650201	NT	>50	>50	>50	>50	>50

				maternal viruses						
	Specificity	Sample	SVA.MulV	5807m2	5807m3	5807m15	5807m17	5807m18	5807m20	5807m30
5807	polyclonal	plasma	<20	<20	<20	<20	<20	41	48	26
	V3-specific	H650207	NT	>50	>50	>50	>50	>50	34.75	>50
	V3-specific	H650211	NT	>50	>50	>50	>50	>50	39.74	>50
	CD4bs-specific	H650215	NT	>50	>50	>50	>50	>50	>50	>50
	CD4bs-specific	H650218	NT	>50	>50	>50	>50	>50	>50	>50
	V3-specific	H650223	NT	>50	>50	>50	>50	>50	>50	>50
	V1V2-specific	H650226	NT	>50	>50	>50	>50	>50	>50	>50
	V3-specific	H650230	NT	>50	>50	>50	>50	>50	>50	>50
	V1V2-specific	H650237	NT	>50	>50	>50	>50	>50	>50	>50
	V1V2-specific	H650238	NT	>50	>50	>50	>50	>50	>50	>50
	V3-specific	H650239	NT	>50	>50	>50	>50	>50	21.36	>50
	V3-specific	H650241	NT	>50	>50	>50	>50	>50	>50	>50
	V3-specific	H650249	NT	>50	>50	>50	>50	>50	>50	>50
	V3-specific	H650262	NT	>50	>50	>50	>50	>50	22.02	>50
	V3-specific	H650277	NT	>50	>50	>50	>50	>50	11.07	>50
