# Supplementary material for: Maternal Broadly Neutralizing Antibodies Can Select for Neutralization-Resistant, Infant-Transmitted/Founder HIV Variants
Source: mBio. 2020 Mar 10;11(2):e00176-20. doi: 10.1128/mBio.00176-20 (PMC7064758; doi:10.1128/mBio.00176-20)
Supplement: FIG S4 [file mBio.00176-20-sf004.pdf]

Closest to infant T/F virus — Phylogenetic distance → Most distant to infant T/F virus

ID50	IC50 (µg/ml)
<20	>50
<100	21-49.9
>100	5-20.9

NT: not tested

				infant T/F	maternal viruses									
	Specificity	Sample	SVA.MulV	155i21	155m29	155m15	155m18	155m17	00155m9	155m12	155m4	155m33	155m48	155m51
155.1	polyclonal	plasma	<20	21	<20	22	<20	<20	26	<20	<20	22	35.77	<20
	CD4bs-specific	H650013	NT	>50	>50	>50	>50	>50	>50	>50	>50	>50	>50	>50
	CD4bs-specific	H650015	NT	>50	>50	>50	>50	>50	>50	>50	>50	>50	>50	>50
	gp120-specific	H650017	NT	>50	>50	>50	>50	>50	>50	>50	>50	>50	>50	>50
	V3-specific	H650023	NT	>50	>50	>50	>50	>50	>50	>50	>50	>50	>50	>50

				infant T/F	maternal viruses										
	Specificity	Sample	SVA.MulV	0616i1	0601m45	0601m16	0601m36	0601m49	0601m42	0601m21	0601m19	0601m39	0601m50	0601m24	0601m23
0601	polyclonal	plasma	<20	40	24	64	32	149	29	88	31	31	52	52	66
	V1V2-specific	H650032	NT	>50	>50	>50	>50	>50	>50	>50	>50	>50	>50	>50	>50
	V1V2-specific	H650033	NT	>50	>50	>50	>50	>50	>50	>50	>50	>50	>50	>50	>50
	CD4bs-specific	H650035	NT	>50	>50	>50	>50	>50	>50	>50	>50	>50	>50	>50	>50
	V1V2-specific	H650038	NT	>50	>50	>50	>50	>50	>50	>50	>50	>50	>50	>50	>50
	CD4bs-specific	H650065	NT	>50	>50	>50	>50	>50	>50	>50	>50	>50	>50	>50	>50
	CD4bs-specific	H650070	NT	>50	>50	>50	>50	>50	>50	>50	>50	>50	>50	>50	>50
	V1V2-specific	H650071	NT	>50	>50	>50	>50	>50	>50	>50	>50	>50	>50	>50	>50
	V3-specific	H650076	NT	>50	>50	>50	>50	>50	>50	>50	>50	>50	>50	>50	>50
	V3-specific	H650078	NT	>50	>50	>50	>50	>50	>50	>50	>50	>50	>50	>50	>50
	V3-specific	H650081	NT	>50	>50	>50	>50	>50	>50	>50	>50	>50	>50	>50	>50

				infant T/F	maternal	infant T/F	Maternal viruses								infant T/F	Maternal viruses							
	Specificity	Sample	SVA.MulV	3915i1	3902m44	3915i30	3902m23	3902m20	3902m35	3902m32	3902m11	3902m36	3902m46	3902m38	3915i22	390229	3902m31	3902m41	3902m42	3902m12	3902m25	3902m43	3902m5
3902	polyclonal	plasma	<20	55	46	24	24	28	58	90	36	129	126	212	26	35	148	116	142	33	35	133	29
	CD4bs-specific	H650119	NT	>50	>50	>50	>50	>50	>50	>50	>50	>50	>50	>50	>50	>50	>50	>50	>50	>50	>50	>50	>50
	CD4bs-specific	H650122	NT	>50	>50	>50	>50	>50	>50	>50	>50	>50	>50	>50	>50	>50	>50	>50	>50	>50	>50	>50	>50
	V3-specific	H650129	NT	>50	>50	>50	>50	>50	>50	>50	>50	>50	>50	>50	>50	>50	>50	>50	>50	>50	>50	20.36	>50
	gp120-specific	H650142	NT	>50	>50	>50	>50	>50	>50	>50	>50	>50	>50	>50	>50	>50	>50	>50	>50	>50	>50	>50	>50

				infant T/F	maternal viruses												
	Specificity	Sample	SVA.MulV	9112i11	9105m10	9105m20	9105m27	9105m14	9105m7	9105m35	9105m15	9105m6	9105m32	9105m21	9105m17	9105m5	9105m22
9105	polyclonal	plasma	44	185	63	73	85	51	116	48	48	62	103	120	54	56	86
	gp120-specific	H650088	NT	>50	>50	>50	>50	>50	>50	>50	>50	>50	>50	>50	>50	>50	>50
	V3-specific	H650090	NT	>50	>50	>50	>50	>50	>50	>50	>50	>50	>50	>50	>50	>50	>50
	CD4bs-specific	H650091	NT	>50	>50	>50	>50	>50	>50	>50	>50	>50	>50	>50	>50	>50	>50
	CD4bs-specific	H650095	NT	>50	>50	>50	>50	>50	>50	>50	>50	>50	>50	>50	>50	>50	>50
	V3-specific	H650099	NT	>50	>50	>50	>50	>50	>50	>50	>50	>50	28.57	>50	>50	>50	>50
	gp120-specific	H650104	NT	>50	>50	>50	>50	>50	>50	>50	>50	>50	>50	>50	>50	>50	>50
	gp120-specific	H650108	NT	>50	>50	>50	>50	>50	>50	>50	>50	>50	>50	>50	>50	>50	>50
	gp120-specific	H650112	NT	>50	>50	>50	>50	>50	>50	>50	>50	>50	>50	>50	>50	>50	>50
	CD4bs-specific	H650114	NT	>50	>50	>50	>50	>50	>50	>50	>50	>50	>50	>50	>50	>50	>50
