# Supplementary material for: Maternal Broadly Neutralizing Antibodies Can Select for Neutralization-Resistant, Infant-Transmitted/Founder HIV Variants
Source: mBio. 2020 Mar 10;11(2):e00176-20. doi: 10.1128/mBio.00176-20 (PMC7064758; doi:10.1128/mBio.00176-20)

A

V3	NNTRKSIHIGPGRAFYATGDIIGDIRQAHC
V3 K305Q/I307T/H308T	NNTRQSTTIGPGRAFYATGDIIGDIRQAHC
V3 F317L/A319T/D322R	NNTRKSIHIGPGRALYTT-RIIGDIRQAHC
V3 F317A/A319K/D322A	NNTRKSIHIGPGRAAYKTGAIIGDIRQAHC
V3 K305A	NNTRASIHIGPGRAFYATGDIIGDIRQAHC
V3 I307A	NNTRKSAHIGPGRAFYATGDIIGDIRQAHC
V3 H308A	NNTRKSIAIGPGRAFYATGDIIGDIRQAHC
V3 F317A	NNTRKSIHIGPGRAAYATGDIIGDIRQAHC
V3 A319K	NNTRKSIHIGPGRAFYKTGDIIGDIRQAHC
V3 D322A	NNTRKSIHIGPGRAFYATGAIIGDIRQAHC

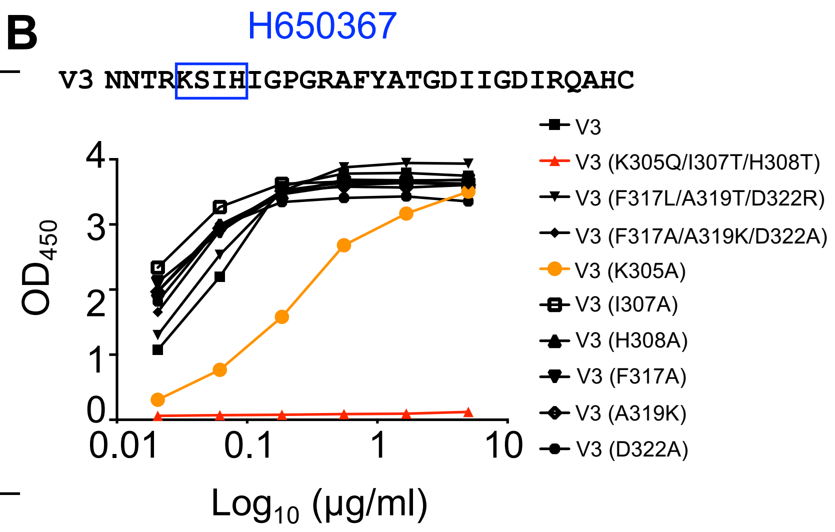

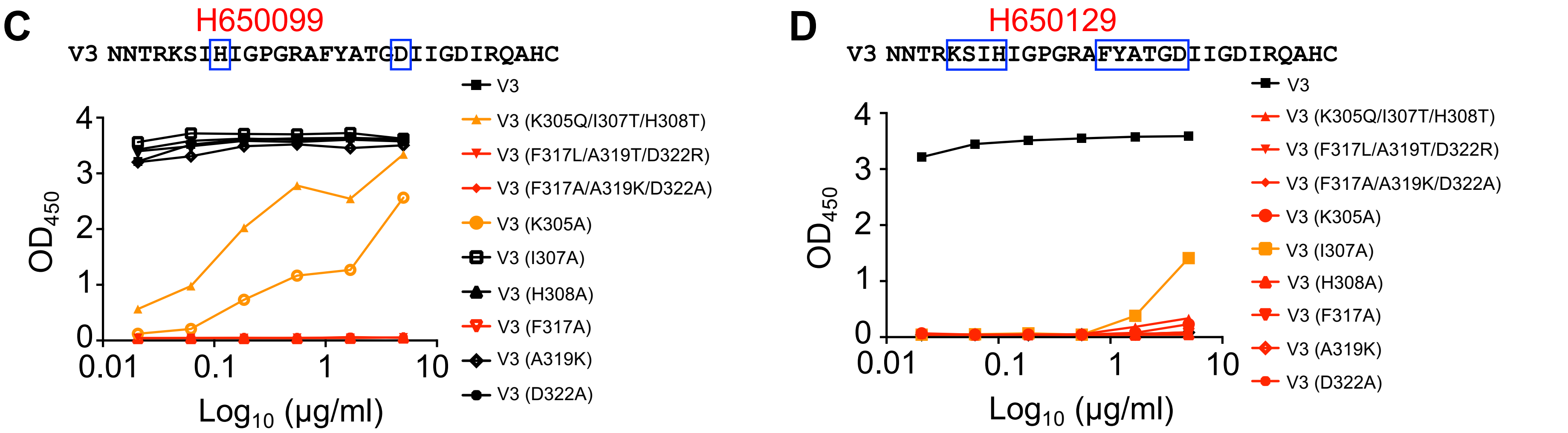

Supplement: FIG S5 [file mBio.00176-20-sf005.pdf]
