# Supplementary material for: Maternal Broadly Neutralizing Antibodies Can Select for Neutralization-Resistant, Infant-Transmitted/Founder HIV Variants
Source: mBio. 2020 Mar 10;11(2):e00176-20. doi: 10.1128/mBio.00176-20 (PMC7064758; doi:10.1128/mBio.00176-20)
Supplement: FIG S6 [file mBio.00176-20-sf006.pdf]

A

PTID/mAb	Ce1176	Ce1176 N276Q	Ce1176 N88A	Ce1176 N625A	Ce1176 N332A
3902	1122	726	846	969	1030
PGT128	<0.02	<0.02	NT	NT	24.5
VRC01	3.93	1.49	4.04	4.73	5.12
35022	0.03	0.02	>50	>50	NT

↑ wild type (Ce1176)
↑ CD4bs mutant (Ce1176 N276Q)
gp120/gp41 interface mutants (Ce1176 N88A, Ce1176 N625A)
↑ V3 glycan mutant (Ce1176 N332A)

ID_{50}	IC_{50} (μg/ml)
<20	>50
21-100	21-49.9
101-200	5-20.9
201-500	1.1-4.99
>501	<0.02-1

NT: not tested
