# Supplementary material for: Maternal Broadly Neutralizing Antibodies Can Select for Neutralization-Resistant, Infant-Transmitted/Founder HIV Variants
Source: mBio. 2020 Mar 10;11(2):e00176-20. doi: 10.1128/mBio.00176-20 (PMC7064758; doi:10.1128/mBio.00176-20)

A

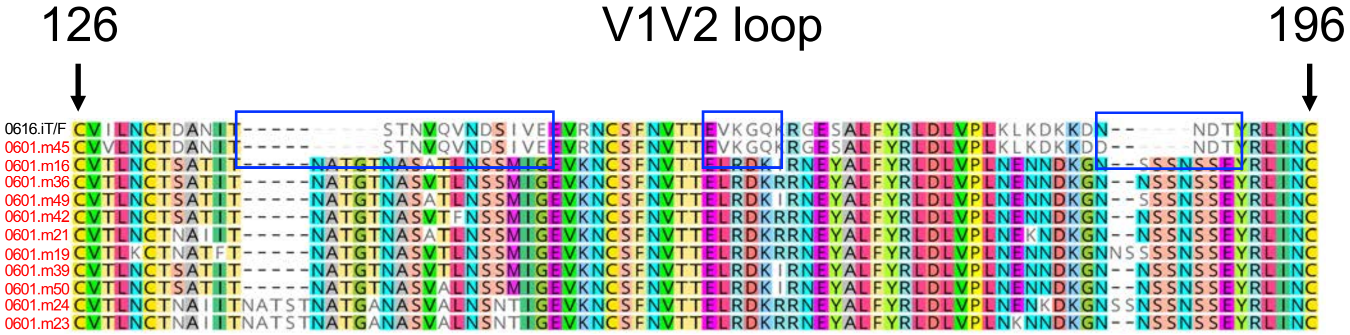

B

PTID	246F3 WT	246F3 N160K	BJOX2000 WT	BJOX2000 N160K	Ce1176 WT	Ce1176 N160K	X1632 WT	X1632 N160K	Ce7030702 WT	Ce7030702 N160K	25710 WT	25710 N160K	CH119 WT	CH119 N160A
0601	25	34	<20	104	23	21	<20	<20	<20	24	40	60	28	27
PG9	<0.02	>50	0.07	>50	<0.02	>50	0.05	>50	<0.02	>50	<0.02	>50	0.41	>50
PGT128			0.03	0.03										
VRC01	0.32	0.55			2.25	4.16	0.12	0.06	0.3	0.29	0.6	0.56	1.69	0.95
	wild type	V2 glycan mutant	wild type	V2 glycan mutant	wild type	V2 glycan mutant	wild type	V2 glycan mutant	wild type	V2 glycan mutant	wild type	V2 glycan mutant	wild type	V2 glycan mutant

C

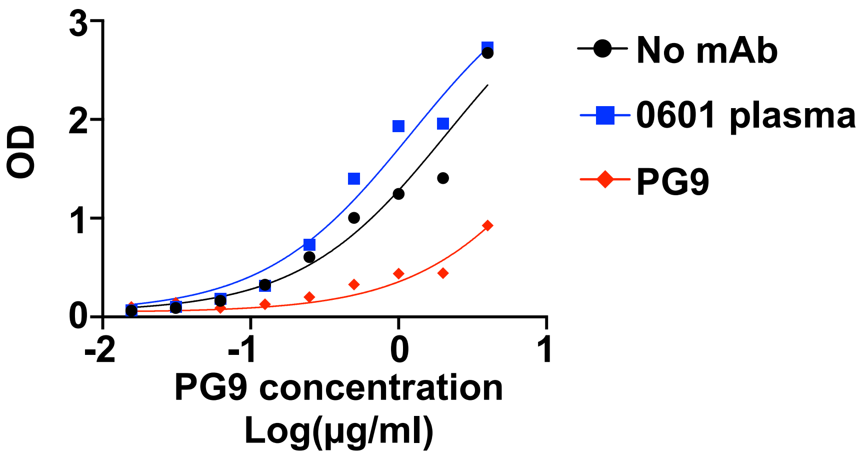

ID50	IC50 (µg/ml)	NT: not tested
<20	>50	
21-100	21-49.9	
101-200	5-20.9	
201-500	1.1-4.99	
>501	<0.02-1	

D

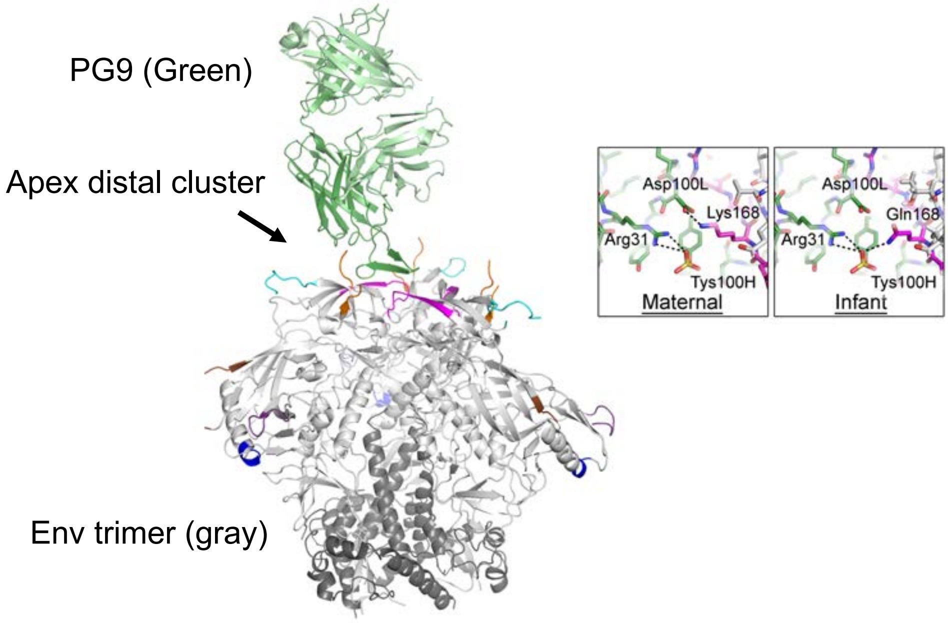

Supplement: FIG S7 [file mBio.00176-20-sf007.pdf]
