# Supplementary material for: Maternal Broadly Neutralizing Antibodies Can Select for Neutralization-Resistant, Infant-Transmitted/Founder HIV Variants
Source: mBio. 2020 Mar 10;11(2):e00176-20. doi: 10.1128/mBio.00176-20 (PMC7064758; doi:10.1128/mBio.00176-20)
Supplement: FIG S8 [file mBio.00176-20-sf008.pdf]

A

Amino acid residue position	732	PG9 IC_{50} (µg/ml)
HXB2 residue	G	NT
9105m.10	G	>50
9105m.20	G	>50
9105m.27	G	>50
9105m.14	G	>50
9105m.7	G	>50
9105m.35	G	>50
9105m.15	G	>50
9105m.6	G	>50
9105m.32	G	>50
9105m.21	G	>50
9105m.17	G	>50
9105m.5	G	>50
9105m.22	G	>50

IC_{50} (µg/ml) **NT: not tested**

>50
21-49.9
5-20.9
1.1-4.99
<0.02-1

B

Amino acid residue position	33	169	633	644	775	PG9 IC_{50} (µg/ml)
HXB2 residue	K	V	R	S	L	NT
0155m.29	Q	M	A	T	L	0.18
0155m.15	K	V	R	N	I	>50
0155m.18	K	V	R	N	I	12.09
0155m.17	K	V	R	N	I	>50
0155m.9	K	V	R	N	I	>50
0155m.12	K	V	R	N	L	>50
0155m.4	K	V	R	N	L	>50
0155m.33	Q	M	A	T	L	0.08
0155m.48	Q	M	A	T	L	>50
0155m.51	Q	M	A	T	L	0.08
